# Photoperiodic Effects on Diurnal Rhythms in Cell Numbers of Peripheral Leukocytes in Domestic Pigs

**DOI:** 10.3389/fimmu.2019.00393

**Published:** 2019-03-12

**Authors:** Larissa C. Engert, Ulrike Weiler, Birgit Pfaffinger, Volker Stefanski, Sonja S. Schmucker

**Affiliations:** Behavioral Physiology of Livestock, Institute of Animal Science, University of Hohenheim, Stuttgart, Germany

**Keywords:** photoperiod, season, diurnal rhythm, circadian rhythm, immune system, cortisol, activity behavior, swine

## Abstract

The photoperiod is known to modulate immune cell number and function and is regarded essential for seasonal disease susceptibility. In addition, diurnal variations in the immune system are regarded important for immune competence. Whereas few studies investigated the influence of season, none investigated the specific effect of the photoperiod on these diurnal immune rhythms until now. Therefore, the present study compared diurnal rhythms in cell numbers of peripheral leukocyte types in domestic pigs held either under long day conditions (LD) or short day conditions (SD). Cosinor analyses of cell numbers of various peripheral leukocyte subtypes investigated over periods of 50 h revealed distinct photoperiodic differences in diurnal immune rhythms. Relative amplitudes of cell numbers of total leukocytes, NK cells, T cells, and monocytes in blood were higher under SD than LD. In addition, cell counts of total leukocytes, NK cells, T cells including various T cell subtypes, and eosinophils peaked earlier relative to the time of lights-on under SD than LD. In contrast, diurnal rhythms of neutrophil counts did not show photoperiodic differences. Mesor values did not differ in any leukocyte type. Generalized linear mixed model analyses revealed associations of leukocyte counts with plasma cortisol concentration and activity behavior in most investigated cell types. Moreover, the present study demonstrated photoperiodic effects on diurnal rhythms in plasma cortisol concentrations and activity behavior, which is in agreement with human and primate studies. The results of the present study imply stronger rhythmicity in leukocyte counts in general under SD. Common intrinsic mechanisms seem to regulate photoperiodic effects on diurnal rhythms in leukocyte counts, except for neutrophils, in domestic pigs. Our results reveal considerable insights into the regulation of immune rhythms in diurnally active species.

## Introduction

Diurnal and seasonal rhythms are important characteristics of physiology and behavior in humans and animals (1–4). Thereby, the alteration between day and night regulates diurnal (i.e., 24 h) rhythmicity via photic entrainment of the master circadian clock located in the *suprachiasmatic nucleus* (SCN) within the anteroventral hypothalamus of the brain (5–7). Correspondingly, seasonal rhythms are assumed to be mediated by an intrinsic circannual clock as well, potentially located in the *pars tuberalis* within the anterior pituitary (8–10), with the relative span of light per day (i.e., long photoperiod during summer, short photoperiod during winter) serving as seasonal timer (4).

Beside differences in physiology and behavior, seasonal differences were also found in the incidence of disease and mortality in many species (11–19). In this respect, the photoperiod is regarded essential for seasonal disease susceptibility as it is known to modulate immune function (15, 20–23). Seasonal differences in the mammalian immune system were already described in humans and rodent models, whereas photoperiodic effects in particular were investigated in rodents only (21, 23–27). In addition to seasonal differences, diurnal variations in the immune system are well-documented in humans and rodents and are regarded important for immune competence due to timely orchestration of immune function (28–30). Only few studies investigated seasonal modulations of diurnal rhythms in the mammalian immune system (31–36) and to our knowledge, none investigated the specific effect of the photoperiod on diurnal immune rhythms until now. Moreover, whereas important mediators of diurnal rhythmicity in the immune system, such as glucocorticoids and the sympathetic nervous system, were already identified (37, 38), mechanisms driving seasonal changes in the immune system are not clearly defined yet, especially in diurnally active species.

Our group recently demonstrated the occurrence of diurnal rhythms in peripheral immune cell numbers in the diurnally active domestic pig (39), which is regarded as highly suitable model species as it provides great anatomical, physiological, and immunological similarity with humans (40). In addition, studying mechanisms of seasonal disease susceptibility in this species may result in improvement of animal health and welfare within pig husbandry systems.

The present study, therefore, investigated photoperiodic effects on diurnal rhythms in immune cell numbers of particular leukocyte types in domestic pigs. We assessed diurnal rhythmicity of cell numbers in various immune cell types as well as plasma cortisol concentration, activity behavior, and hematocrit of pigs held under two different lighting regimes with cosinor analysis (41) and performed generalized linear mixed model analysis to evaluate potential associations between the investigated variables.

## Materials and Methods

### Animals, Experimental Conditions, and Surgery

All procedures were conducted in accordance with the German Animal Welfare Act and approved by the local Animal Welfare Ethics Committee (Regional Council Stuttgart, approval number V309/13TH). A total of 20 castrated male pigs (*Sus scrofa domestica*, Piétrain × German landrace, 7- to 8-month-old, body weight range 104–131 kg) were included in the study, subdivided into four different randomized experimental trials, which were conducted between April and May in 2015 and 2016. The pigs were housed in a lightproof building of the experimental unit of the department Behavioral Physiology of Livestock at a constant ambient temperature of 21 ± 1°C. The building is subdivided into two units, each equipped with an autonomous lighting system enabling different lighting regimes in parallel. Within every trial, one portion of animals was held under long day conditions (LD) with a photoperiod of 16 h per day (16L:8D, lights on 07:00–23:00, *n* = 9 pigs) and the other portion of animals was held under short day conditions (SD) with a photoperiod of 8 h per day (8L:16D, lights on 07:00–15:00, *n* = 11 pigs). The average illuminance was 190 lx at pigs' eye level during the light phase (fluorescent tubes, Philips Master TL-D Super 80 58W/840, color temperature 4,000 K) and 0 lx during the dark phase. Allocation of pigs to LD or SD treatment was performed randomly and balanced for littermates. They were kept in individual pens (6.4 m^2^ each) with sight and tactile contact to neighboring animals. All animals had *ad libitum* access to hay and water and were fed concentrate twice daily at 07:30 and 14:00 (1.1–1.2 kg/meal, ME 12 MJ/kg). Pens were cleaned and littered with dust-free wood shavings daily after concentrate feeding in the morning. All pigs were accustomed to the respective lighting and feeding regime for at least 4 weeks prior to the first sampling after being held for habituation under 12L:12D lighting conditions (lights-on at 07:00) for at least 6 weeks. Within this habituation period, time was switched from Central European Time (CET) to Central European Summer Time (CEST) at least 2 weeks prior to introduction of LD or SD. To enable blood collection without disturbing the animals, all pigs were well-habituated to human handling and surgically catheterized with indwelling vein catheters (introduced into the *vena cava cranialis* via the *vena cephalica*) at least 2 weeks prior to the experiments as described previously (39). Catheters were rinsed with heparinized saline (115 IU/ml, heparin sodium salt, Carl Roth, Karlsruhe, Germany) twice a day after concentrate feeding and all animals were weighed once per week (not during sampling periods). Health was monitored by daily measurement of rectal temperature.

### Sampling Protocol and Sample Processing

**Figure 1:**
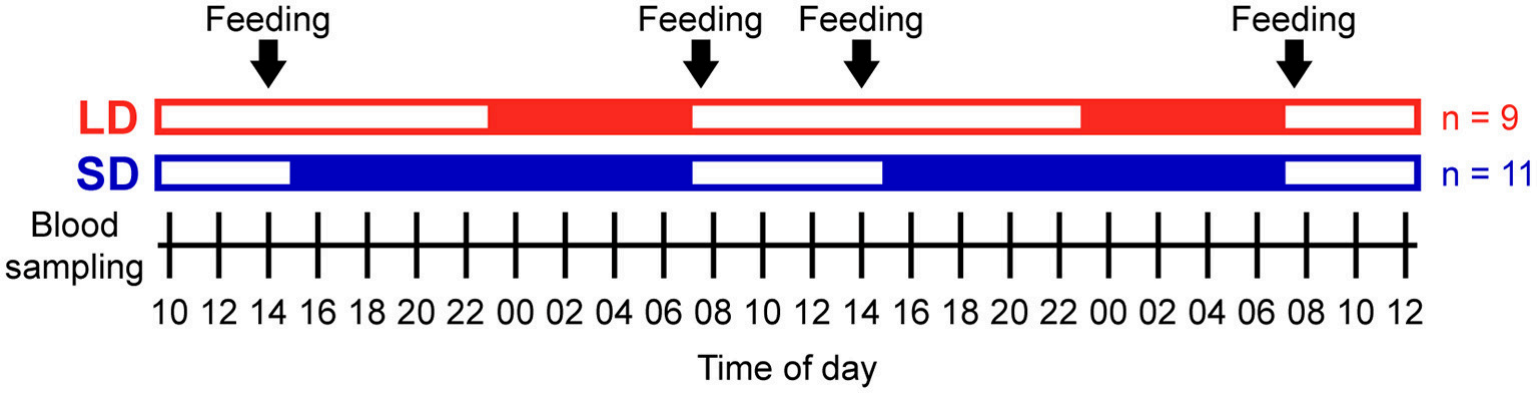
Experimental protocol. Open bars indicate light periods, filled bars indicate dark periods, and arrows indicate concentrate feeding times. A total of nine pigs were studied under long day conditions (LD, red) and a total of 11 pigs were studied under short day conditions (SD, blue) in 4 different randomized trials (*n* = 4 or 6 each). Blood sampling started at 10:00 and was repeated every 2 h over periods of 50 h in each trial.

From each pig, blood samples were taken every 2 h, starting at 10:00 over periods of 50 h (Figure 1, 26 blood samples per animal). At lights-off, the sampling procedure was performed under dim light of averagely 7 lx at pigs' eye level, which was switched on and off for sampling (Philips energy-saving/LED bulbs 3W, color temperature 2,700 K). Sampling all animals lasted not longer than 20 min in total per sampling and animals were sampled in the same order each time. After discarding the heparinized saline solution from the catheters, 10 ml blood per sample was drawn. Subsequently, the catheter was rinsed with ~10 ml heparinized saline (46 IU/ml) to keep the catheter patent and to compensate for the blood volume taken. Blood was transferred directly into lithium heparin tubes and K3 EDTA tubes (both Sarstedt, Nümbrecht, Germany). Blood samples were immediately processed after each sampling.

### Hematology

Total leukocyte counts and hematocrit were analyzed in K3 EDTA blood samples by an automated hematology analyzer (MEK-6108G, Nihon Kohden, Rosbach, Germany). All samples were measured in duplicate. The mean intra-assay coefficient of variation for biological samples was 1.1% for leukocyte counts and 1.0% for hematocrit. Validity was verified by daily reference measurements of a hematology control (Para 12 Extend N, Streck, Omaha, NE, USA). Hematology analyses were finished within 60 min after blood sampling.

### Flow Cytometry

Characterization of specific immune cell populations was performed using three-color flow cytometry with preceding immunofluorescent antibody staining of heparinized whole blood following an established standard protocol using different combinations of monoclonal antibodies (all SouthernBiotech, Birmingham, AL, USA) specific for porcine CD3ε (clone PPT3), CD4 (clone 74-12-4), CD8α (clone 76-2-11), and CD172a (clone 74-22-15) as described previously (39). All antibodies were titrated in preceding experiments for optimal staining. Controls included unlabeled cells, single antibody stains, and fluorescence minus one (FMO) controls. Identification of peripheral blood mononuclear cells (PBMC) and granulocytes was performed based on their forward and side scatter characteristics. Subsequently, non-fluorescent neutrophils were distinguished from autofluorescent eosinophils within granulocytes in an unstained sample (42). Further characterization of immune cells was carried out within PBMC based on surface marker expression following previous reports (43–47): T cells (CD3^+^), naive T helper (Th) cells (CD3^+^ CD4^+^ CD8α^−^), Ag-experienced (Ag-exp.) Th cells (CD3^+^ CD4^+^ CD8α^+^), cytotoxic T cells (CD3^+^ CD4^−^ CD8α^high^), γδ T cells (CD3^+^ CD4^−^ CD8α^−/dim^), NK cells (CD3^−^ CD8α^+^ CD172a^−^), and monocytes (CD3^−^ CD8α^−^ CD172a^high^). The mean intra-assay coefficient of variation was 0.9% for PBMC, 2.1% for granulocytes, and 1.1% for T cells. Results concerning CD8α^−^ and CD8α^+^ γδ T cells found in the present study are presented in Figures S4, S5, Tables S1–S8, as it is not yet known whether the two investigated different phenotypic subsets of porcine γδ T cells also display different functions (44, 48).

### Cortisol Radioimmunoassay

Blood plasma was obtained from heparinized blood samples by centrifugation (15 min at 2,000 × g at 4°C) within 45 min after sampling and stored at −80°C until measurement. Plasma cortisol concentrations were analyzed radioimmunologically with preceding ethanolic extraction as reported previously (39, 49). Briefly, plasma samples were diluted 10-fold with 100% ethanol (Carl Roth), mixed, incubated for 5 min at 4°C, and centrifuged for 10 min at 2,000 × g at 4°C to remove binding proteins. Aliquots of ethanolic supernatant were evaporated to dryness, resuspended in phosphate buffer, and analyzed in the RIA in duplicate. Therefore, a polyclonal antibody against cortisol-3-BSA (MBS316242, MyBioSource, San Diego, CA, USA) at a final dilution of 1:112,000 in 0.1% BSA buffer was added and [1,2-^3^H]-cortisol (50 Ci/mmol, American Radiolabeled Chemicals, Saint Louis, MO, USA) or [1,2,6,7-^3^H]-cortisol (93 Ci/mmol, PerkinElmer, Boston, MA, USA) was used as tracer. Separation of bound/free was performed with dextran-coated charcoal (0.02% Dextran 70, Carl Roth; 0.2% Norit A, Serva Electrophoresis, Heidelberg, Germany) by centrifugation for 20 min at 2,000 × g at 4°C. Supernatants were decanted to 5 ml Irga-Safe Plus (PerkinElmer) to determine radioactivity. Precision was determined with spiked controls and revealed a mean recovery rate of 105%. Intra-assay and inter-assay variabilities for biological samples were 2.7% and 8.3% (33.2 ng/ml) as well as 10.0% and 13.9% (16.2 ng/ml), respectively.

### Activity Behavior

Animal behavior was recorded with cameras and analyzed as described previously (39). In brief, recording started 2 h before the first blood sampling and was continued throughout each sampling period. The software *The Observer XT 11* (Noldus Information Technology, Wageningen, The Netherlands) was used to categorize animal behavior by focal sampling and continuous recording into inactivity or activity. Thereby, resting of pigs in prone or lateral position was classified as inactivity, whereas all other behaviors were classified as activity. Relative activity behavior was calculated for every single animal as proportion of time in which the animal was active within each 2-h interval preceding the respective blood sampling.

### Statistical Analyses

Diurnal rhythmicity was assessed by cosinor analysis (41) using the package *cosinor* (50) in R version 3.1.2 (R Foundation for Statistical Computing, Vienna, Austria). Period length was set to 24 h in all cosinor models. Overall diurnal rhythmicity was investigated by performing cosinor analyses with combined datasets of all animals per treatment group. Subsequently, individual diurnal rhythmicity was assessed by rerunning cosinor analyses for every single animal in the study (refer to Table S1). Diurnal rhythms were characterized by mesor (average value of the fitted cosine function), amplitude (half the difference between maximum and minimum of the fitted cosine function), and peak time (time of the maximum of the fitted cosine function). In addition, the phase distribution, defined as standard deviation of mean peak times (51), was calculated from individual cosinor analysis results as a measure of peak time dispersal. Diurnal rhythms were considered significant if cosinor models revealed *P* < 0.05 for the amplitude. Peak times were calculated by the formula –Φ24/(2π) using the phase shift Φ denoted by R and by setting 00:00 (24 h) as reference time. For visual evaluation of datasets, diurnal profiles are shown without superimposed cosine curves in Figure S5.

Comparisons of mesor values, relative amplitudes, and peak times were performed with IBM SPSS Statistics 22 (IBM Deutschland, Ehningen, Germany). For pairwise comparisons the assumptions of normality and homogeneity of variances were checked with Shapiro–Wilk tests and Levene's tests, respectively. If data met these assumptions, they were compared with two-tailed, unpaired Student's *t*-tests. Otherwise, two-tailed unequal variance *t*-tests (lack of variance homogeneity) or two-tailed Mann–Whitney *U*-tests (both assumptions violated) were used. In the case of paired Student's *t*-tests, normality of differences was confirmed by Shapiro–Wilk tests. For multiple comparisons, normality of data in all groups was analyzed with Shapiro–Wilk tests. If normal distribution was confirmed, repeated measures ANOVA with Mauchly's test to check sphericity was performed. Greenhouse-Geisser correction was applied if sphericity was not met. Bonferroni correction was used for multiple pairwise *post-hoc* testing. If data were not normally distributed, Friedman test was performed. Subsequent Wilcoxon signed-rank tests were conducted either with Bonferroni–Holm correction or with Benjamini–Hochberg correction to compare five groups (52). Two-way factorial effects were investigated with 2 × 2 mixed factorial ANOVA (normality of data confirmed by Shapiro–Wilk tests) followed by inspection of simple main effects with the least significant difference (LSD) procedure if interactions were significant.

Potential associations between the cell numbers of the investigated immune cell populations in porcine blood with experimental factors and different intrinsic variables were assessed using IBM SPSS Statistics 22 to analyze the data of all animals (repeated measurements) with generalized linear mixed models (GLMM, GENLINMIXED command, Satterthwaite's approximation to calculate denominator degrees of freedom). The residual pseudo likelihood approach (REPL) was used for parameter estimation. In all models, the intercept, the factors treatment (LD/SD), light (off/on, indicating darkness or light), and concentrate feeding (yes/no, indicating the samplings at or just after concentrate feeding or not) as well as the covariates plasma cortisol concentration (ng/ml), relative activity behavior (%), hematocrit (%), and sampling (1–26, indicating the ongoing number of samplings) were set as fixed effects. Sampling was specified as repeated effect with animal identity as subject variable and the residual covariance structure was set as first order autoregressive (AR(1)). The factor animal identity (*n* = 20) was set as random effect with a scaled identity (ID) covariance structure. The most appropriate random model term according to the lowest AIC was selected by exclusion of potential other effects, e.g., experimental trial (*n* = 4), dam (*n* = 10), or sire (*n* = 7), by backward model selection on total leukocyte counts only. The final model structure was applied to all other immune cell populations to achieve comparability among them. All models were calculated in six different specifications: Normal distribution with identity or log link functions, inverse Gaussian distribution with identity or log link functions, or gamma distribution with identity or log link functions. For each immune cell type, the most appropriate model structure was identified by evaluation of homoscedasticity and normality by plotting residuals vs. predicted values and by quantile-quantile plots of residuals, respectively. In all models *P* < 0.05 was considered significant (refer to Table S2 for details).

## Results

### Diurnal Rhythms in Immune Cell Numbers Differ Between LD and SD in Porcine Blood

**Figure 2:**
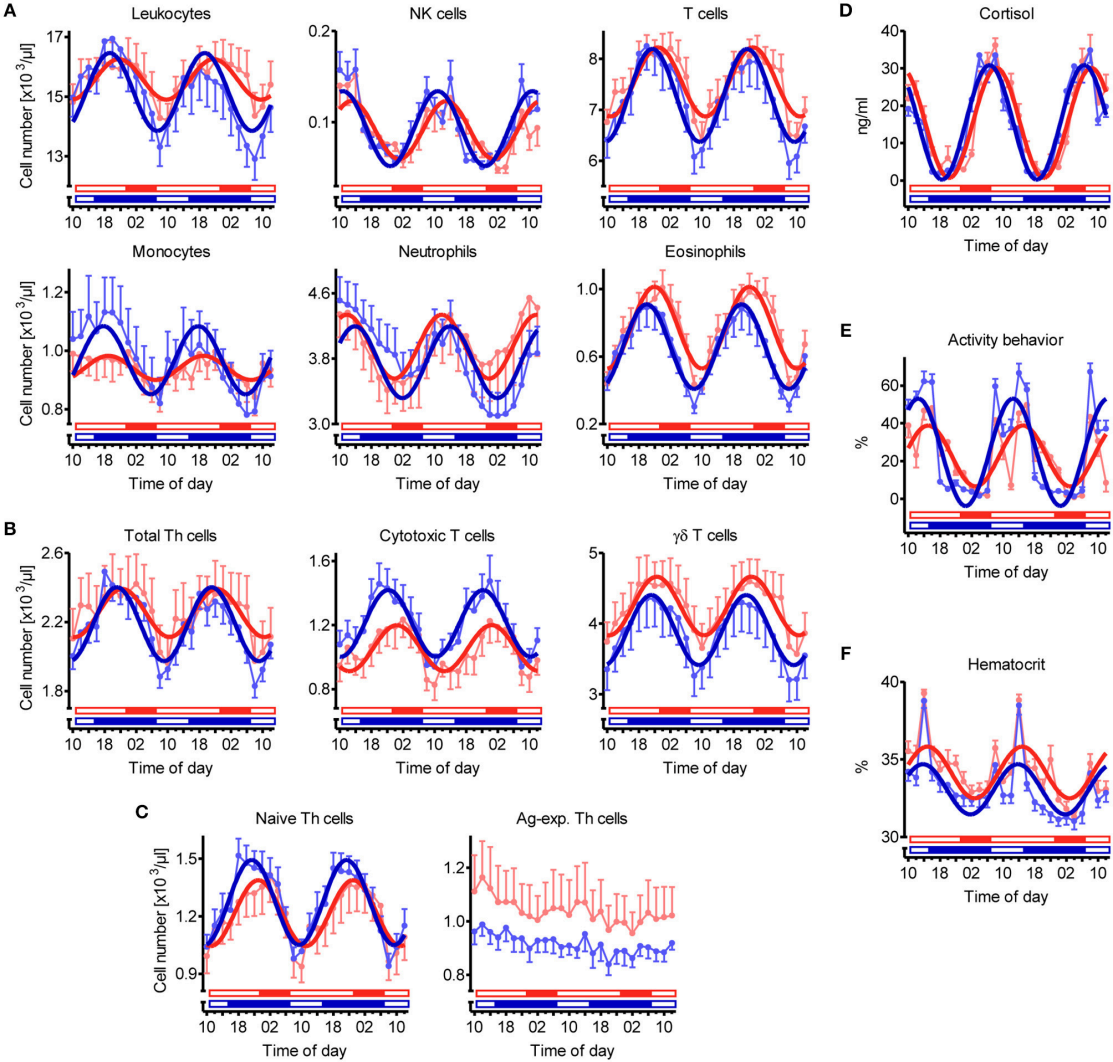
Diurnal rhythms of the cell numbers of different immune cell populations in blood, plasma cortisol concentration, activity behavior, and hematocrit in domestic pigs. Values for pigs held under long day conditions (LD) are shown in red, values for pigs held under short day conditions (SD) are shown in blue. Open bars indicate light periods, filled bars indicate dark periods for the respective lighting regime. Measured values are shown in pale color per treatment (mean ± SEM, LD *n* = 9, SD *n* = 11). Dark curves correspond to the results of overall cosinor analyses with combined datasets of all animals per treatment (LD *n* = 9, SD *n* = 11, significant diurnal rhythm at *P* < 0.05, refer to Table 1). Diurnal rhythms in cell numbers in porcine blood are depicted for **(A)** main immune cell populations, **(B)** different T cell subpopulations, and **(C)** Th cell subtypes with distinctive differentiation states. In addition, diurnal rhythms in **(D)** plasma cortisol concentration, **(E)** activity behavior (values represent the proportion of time, in which the animals were active within the 2-h interval preceding blood sampling), and **(F)** hematocrit are shown.

Overall cosinor analyses with combined datasets of all animals per treatment revealed diurnal rhythmicity (*P* < 0.05) in immune cell numbers in blood under both lighting regimes for total leukocytes, NK cells, T cells, monocytes, neutrophils, and eosinophils, as well as for the majority of investigated T cell subtypes, i.e., total Th cells, cytotoxic T cells, γδ T cells, and naive Th cells (Table 1, Figures 2A–C). Ag-exp. Th cell counts in porcine blood did not show overall diurnal rhythmicity (*P* ≥ 0.05) under LD nor under SD (Table 1, Figure 2C). Individual cosinor analyses per animal revealed diurnal rhythms (*P* < 0.05) in the cell numbers of NK cells, T cells, eosinophils, cytotoxic T cells, γδ T cells, and naive Th cells in blood in all animals under both lighting regimes (Table S1). The cell numbers of total leukocytes, monocytes, and total Th cells in blood exhibited diurnal rhythmicity (*P* < 0.05) in all animals held under SD but only in a proportion of animals under LD, whereas neutrophil counts exhibited diurnal rhythmicity (*P* < 0.05) in all animals held under LD and in a proportion of 91% of animals under SD (Table S1). Finally, Ag-exp. Th cell numbers in blood oscillated diurnally (*P* < 0.05) only in a proportion of animals under both lighting regimes (Table S1).

**Table 1:** Results of overall cosinor analyses with combined datasets of all animals held under long day conditions (LD, *n* = 9) or short day conditions (SD, *n* = 11).

**Variable**	**Treatment**	***P*^a^**	**Mesor**	**Amplitude**	**Amplitude [%]^b^**	**Peak time^c^**
Leukocytes [/μl]	LD	<0.001	15577.8 ± 131.3	678.9 ± 181.2	4.4 ± 1.2	21:37 ± 01:04
	SD	<0.001	15156.1 ± 163.4	1303.6 ± 233.4	8.6 ± 1.5	19:20 ± 00:40
NK cells [/μl]	LD	<0.001	91.5 ± 3.2	31.8 ± 4.5	34.7 ± 4.9	12:59 ± 00:33
	SD	<0.001	93.1 ± 2.8	41.1 ± 3.8	44.1 ± 4.1	10:47 ± 00:22
T cells [/μl]	LD	<0.001	7548.4 ± 64.5	679.9 ± 88.3	9.0 ± 1.2	22:51 ± 00:32
	SD	<0.001	7283.3 ± 80.7	913.3 ± 111.6	12.5 ± 1.5	21:25 ± 00:29
Monocytes [/μl]	LD	0.006	941.7 ± 10.4	41.1 ± 14.9	4.4 ± 1.6	19:01 ± 01:21
	SD	<0.001	968.0 ± 19.9	116.4 ± 28.9	12.0 ± 3.0	17:43 ± 00:54
Neutrophils [/μl]	LD	0.001	3946.9 ± 81.3	390.6 ± 111.6	9.9 ± 2.8	11:48 ± 01:09
	SD	<0.001	3756.3 ± 58.5	440.8 ± 82.5	11.7 ± 2.2	13:51 ± 00:43
Eosinophils [/μl]	LD	<0.001	770.9 ± 16.9	242.4 ± 23.3	31.4 ± 3.0	21:57 ± 00:23
	SD	<0.001	658.7 ± 17.5	250.8 ± 24.7	38.1 ± 3.7	19:59 ± 00:23
Total Th cells [/μl]	LD	0.003	2252.7 ± 33.8	139.6 ± 46.3	6.2 ± 2.1	22:25 ± 01:21
	SD	<0.001	2187.3 ± 17.3	214.2 ± 24.1	9.8 ± 1.1	21:05 ± 00:27
Cytotoxic T cells [/μl]	LD	<0.001	1055.3 ± 20.5	143.6 ± 28.4	13.6 ± 2.7	00:27 ± 00:47
	SD	<0.001	1210.3 ± 20.5	208.6 ± 28.2	17.2 ± 2.3	22:17 ± 00:33
γδ T cells [/μl]	LD	<0.001	4249.4 ± 63.1	414.9 ± 86.6	9.8 ± 2.0	22:30 ± 00:51
	SD	<0.001	3908.1 ± 69.7	496.9 ± 96.7	12.7 ± 2.5	21:10 ± 00:46
Naive Th cells [/μl]	LD	<0.001	1216.3 ± 22.6	171.7 ± 31.0	14.1 ± 2.5	23:01 ± 00:44
	SD	<0.001	1271.9 ± 16.2	221.8 ± 22.5	17.4 ± 1.8	21:22 ± 00:24
Ag-exp. Th cells [/μl]	LD	0.204	1036.4 ± 22.5	39.9 ± 31.4	3.8 ± 3.0	13:10 ± 03:05
	SD	0.154	915.5 ± 8.8	17.7 ± 12.4	1.9 ± 1.4	13:32 ± 02:43
Cortisol [ng/ml]	LD	<0.001	15.4 ± 0.5	14.7 ± 0.8	95.6 ± 5.0	08:17 ± 00:12
	SD	<0.001	15.5 ± 0.5	15.3 ± 0.7	98.6 ± 4.6	06:31 ± 00:10
Activity behavior [%]	LD	<0.001	22.7 ± 1.2	16.1 ± 1.7	71.0 ± 7.3	14:59 ± 00:23
	SD	<0.001	24.7 ± 1.0	28.4 ± 1.4	115.2 ± 5.8	12:29 ± 00:12
Hematocrit [%]	LD	<0.001	34.2 ± 0.1	1.7 ± 0.2	4.9 ± 0.6	14:49 ± 00:27
	SD	<0.001	33.1 ± 0.1	1.6 ± 0.2	4.9 ± 0.6	13:42 ± 00:26

*Values are presented as mean ± SEM*.

a *Significant diurnal rhythm at P < 0.05*.

b *Relative amplitude (amplitude/mesor)*.

c *Time of day ± hh:mm*.

**Figure 3:**
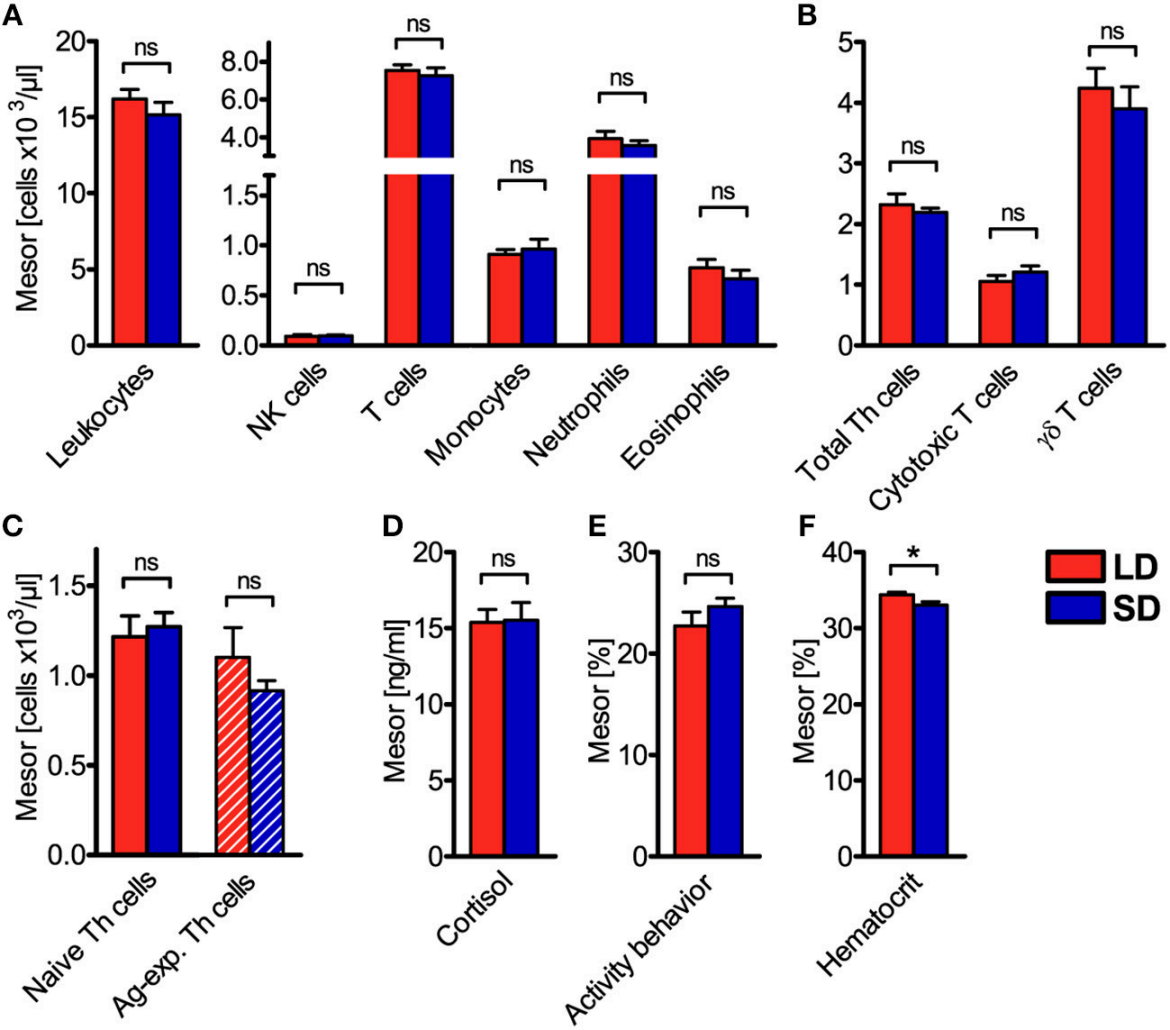
Mesor values of the cell numbers of different immune cell populations in blood, plasma cortisol concentration, activity behavior, and hematocrit in domestic pigs. Values for pigs held under long day conditions (LD) are shown in red, values for pigs held under short day conditions (SD) are shown in blue for **(A)** main immune cell populations, **(B)** different T cell subpopulations, **(C)** Th cell subtypes with distinctive differentiation states, **(D)** plasma cortisol concentration, **(E)** activity behavior, and **(F)** hematocrit. The graphs and statistical analyses only include values of animals with significant (*P* < 0.05) diurnal rhythm in individual cosinor analyses in the respective variable (mean ± SEM, LD *n* = 5–9, SD *n* = 7–11, refer to Table S1). Hatched bars indicate missing diurnal rhythm in overall cosinor analyses with combined datasets of all animals per treatment (significant diurnal rhythm at *P* < 0.05, refer to Table 1). Pairwise comparisons were performed with two-tailed, unpaired Student's *t*-tests (total leukocytes, T cells, monocytes, neutrophils, eosinophils, γδ T cells, plasma cortisol concentration, hematocrit), two-tailed unequal variance *t*-test (activity behavior), or two-tailed Mann–Whitney *U*-tests (NK cells, total Th cells, cytotoxic T cells, naive Th cells, Ag-exp. Th cells); refer to Table S5 for statistical details, ^*^P < 0.05, ns *P* ≥ 0.05.

To assess diurnal rhythmicity between treatments in more detail, the different properties of rhythmicity based on the results of individual cosinor analyses per animal (Table S1) were analyzed. Thereby, mesor values of cell numbers in porcine blood were found not to differ (*P* ≥ 0.05) between LD and SD in any investigated immune cell type (Figures 3A–C). This result was confirmed by calculation of mean values of all 26 measurements per animal, which also did not differ between treatments (Figures S1A–C).

**Figure 4:**
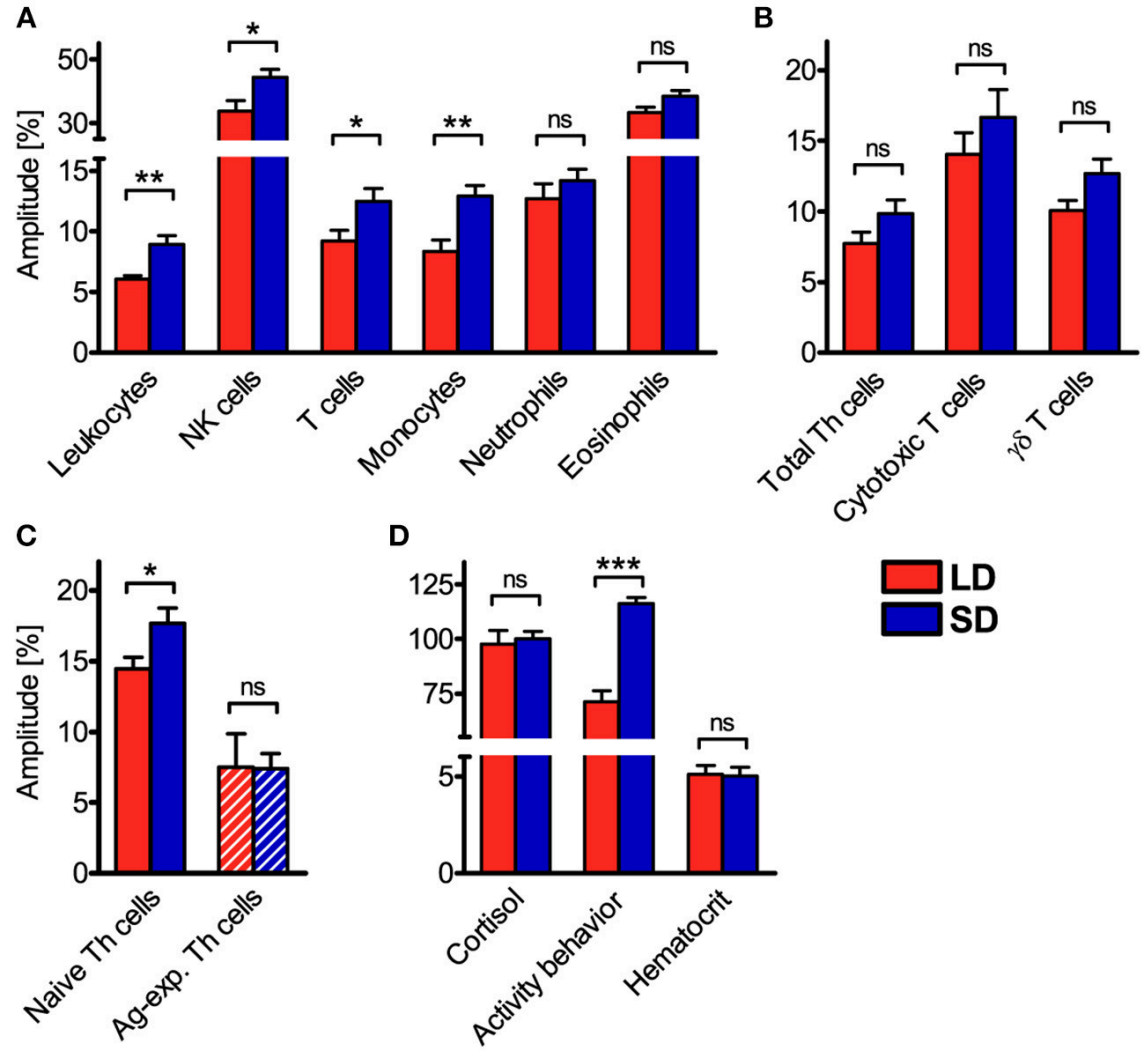
Relative amplitudes of the cell numbers of different immune cell populations in blood, plasma cortisol concentration, activity behavior, and hematocrit in domestic pigs. Values for pigs held under long day conditions (LD) are shown in red, values for pigs held under short day conditions (SD) are shown in blue for **(A)** main immune cell populations, **(B)** different T cell subpopulations, **(C)** Th cell subtypes with distinctive differentiation states, as well as **(D)** plasma cortisol concentration, activity behavior, and hematocrit. The graphs and statistical analyses only include values of animals with significant (*P* < 0.05) diurnal rhythm in individual cosinor analyses in the respective variable (mean ± SEM, LD *n* = 5–9, SD *n* = 7–11, refer to Table S1). Hatched bars indicate missing diurnal rhythm in overall cosinor analyses with combined datasets of all animals per treatment (significant diurnal rhythm at *P* < 0.05, refer to Table 1). Pairwise comparisons were performed with two-tailed, unpaired Student's *t*-tests (NK cells, monocytes, neutrophils, total Th cells, cytotoxic T cells, γδ T cells, activity behavior, hematocrit) or two-tailed Mann–Whitney *U*-tests (total leukocytes, T cells, eosinophils, naive Th cells, Ag-exp. Th cells, plasma cortisol concentration); refer to Table S6 for statistical details, ^***^P < 0.001, ^**^P < 0.01, ^*^P < 0.05, ns *P* ≥ 0.05.

However, relative amplitudes in the cell numbers of total leukocytes, NK cells, T cells, monocytes, and naive Th cells in porcine blood were higher (*P* < 0.05) under SD than LD (Figures 4A,C). In contrast, no difference (*P* ≥ 0.05) in the strength of diurnal oscillation was found between treatments in the cell numbers of neutrophils, eosinophils, total Th cells, cytotoxic T cells, γδ T cells, and Ag-exp. Th cells in porcine blood (Figures 4A–C). Statistical comparisons revealed differences (*P* < 0.05) in the strength of diurnal oscillation among certain immune cell types within each treatment group, which were comparable between LD and SD, with the exception of monocytes (Figure S2).

**Figure 5:**
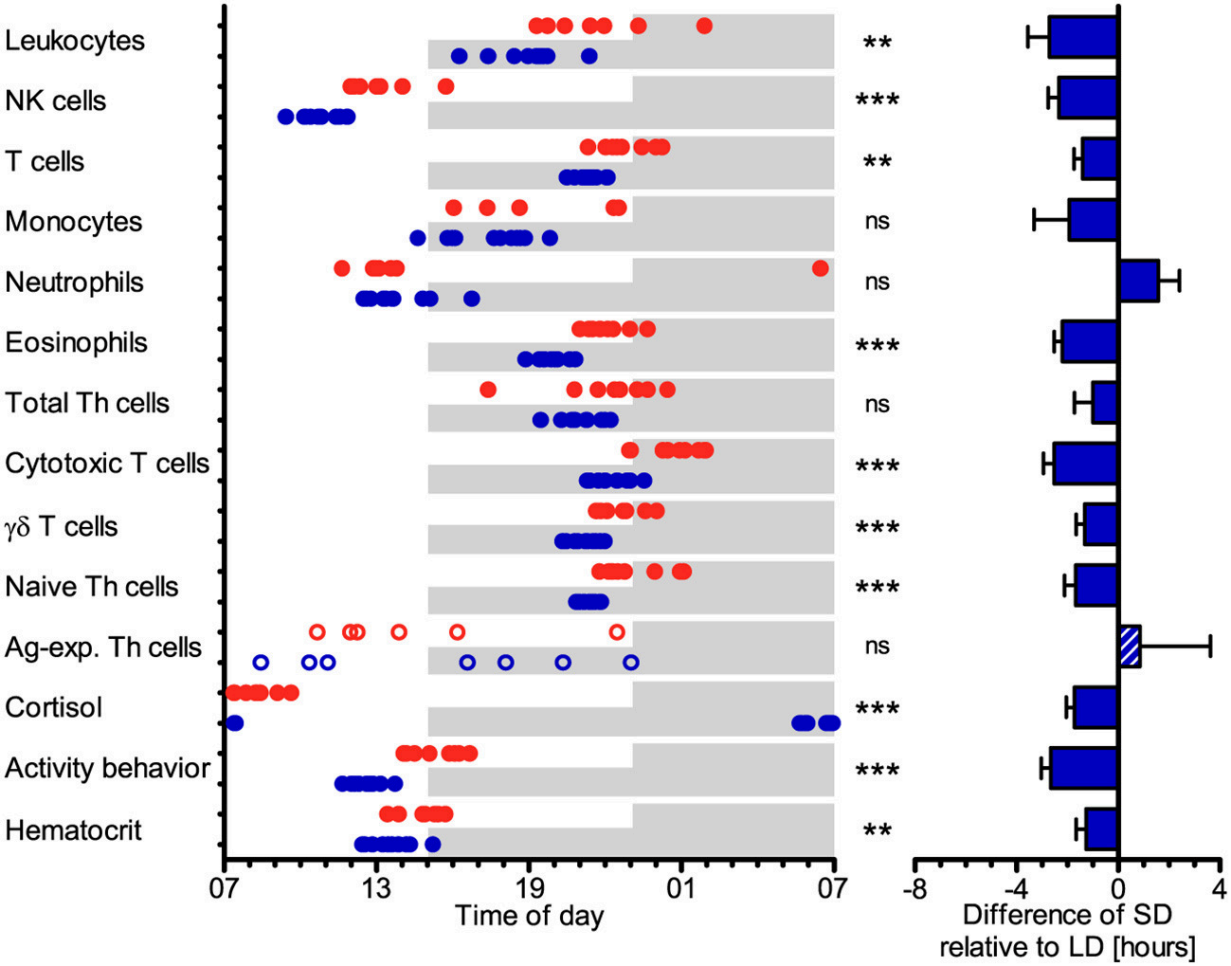
Peak times of the cell numbers of different immune cell populations in blood, plasma cortisol concentration, activity behavior, and hematocrit in domestic pigs. Values for pigs held under long day conditions (LD) are shown in red, values for pigs held under short day conditions (SD) are shown in blue. Shaded areas indicate lights-off in the respective treatments. The graphs and statistical analyses only include values of animals with significant (*P* < 0.05) diurnal rhythm in individual cosinor analyses in the respective variable (LD *n* = 5–9, SD *n* = 7–11, refer to Table S1). The bar graph on the right side illustrates the differences in peak times of SD relative to LD (mean ± SEM). Circles and the hatched bar indicate missing diurnal rhythm in overall cosinor analyses with combined datasets of all animals per treatment (significant diurnal rhythm at *P* < 0.05, refer to Table 1). Pairwise comparisons were performed with two-tailed, unpaired Student's *t*-tests (total leukocytes, NK cells, eosinophils, total Th cells, cytotoxic T cells, γδ T cells, Ag-exp. Th cells, plasma cortisol concentration, hematocrit), two-tailed unequal variance *t*-tests (T cells, monocytes, activity behavior), or two-tailed Mann–Whitney *U*-tests (neutrophils, naive Th cells); refer to Table S7 for statistical details, ^***^P < 0.001, ^**^P < 0.01, ns *P* ≥ 0.05.

The cell numbers of most immune cell types in porcine blood peaked earlier (*P* < 0.05) under SD than LD relative to the time of lights-on at 07:00, i.e., total leukocytes, NK cells, T cells, eosinophils, cytotoxic T cells, γδ T cells, and naive Th cells (Figure 5). The peak times in cell numbers of monocytes, neutrophils, total Th cells, and Ag-exp. Th cells in porcine blood did not differ (*P* ≥ 0.05) between the two lighting regimes (Figure 5). In general, individual peak times in cell counts seem to display greater dispersal under LD than SD in most cell types, except for Ag-exp. Th cells (Table S7). In addition, statistical comparisons of peak times in cell numbers in blood between different leukocyte types within each treatment group revealed differences (*P* < 0.05), which were mainly comparable between LD and SD, with the exception of monocytes and neutrophils (Figure S3).

### Parameters Associated With Immune Cell Numbers in Porcine Blood

In addition to diurnal rhythmicity in immune cell numbers in blood, diurnal rhythms of plasma cortisol concentration, activity behavior, and hematocrit were confirmed (*P* < 0.05) with overall cosinor analyses in combined datasets of all animals per treatment (Table 1, Figures 2D–F). Furthermore, individual cosinor analyses revealed diurnal rhythms (*P* < 0.05) for these variables in almost all animals of the present study (Table S1). The results of individual cosinor analyses revealed that the mesor of hematocrit, in contrast to plasma cortisol and activity behavior, was greater (*P* = 0.036) under LD than SD (Figures 3D–F, Figures S1D–F) and that the relative amplitude of activity behavior, in contrast to cortisol and hematocrit, was higher under SD than LD (Figure 4D). All three variables peaked earlier under SD than LD (Figure 5) and activity behavior seems to exhibit greater peak time dispersal under LD than SD (Table S7).

**Figure 6:**
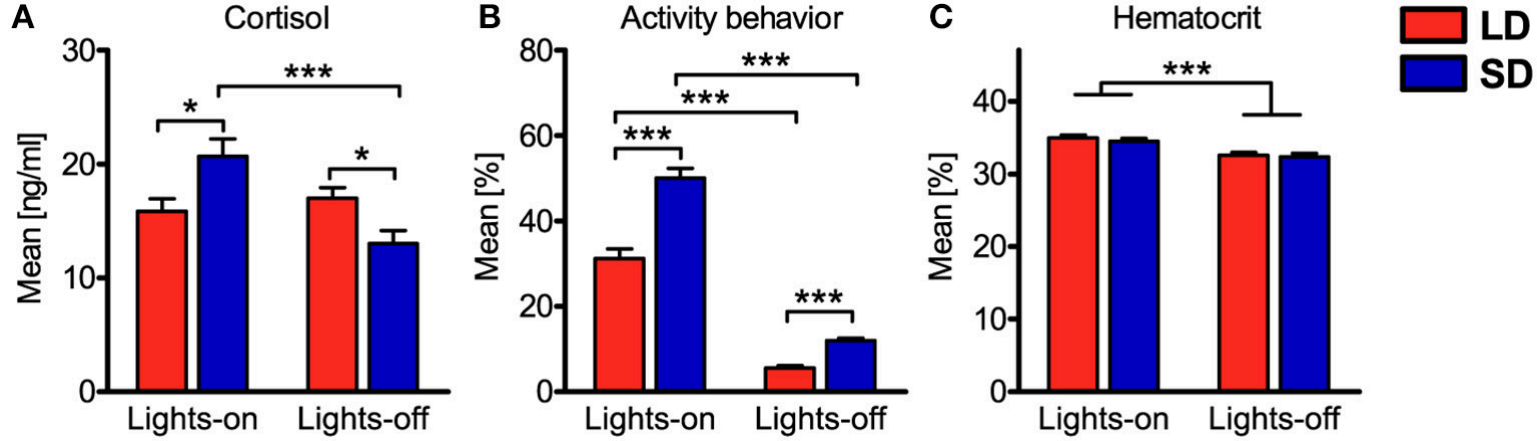
Comparisons of mean values during lights-on or lights-off for the covariates plasma cortisol concentration, activity behavior, and hematocrit. Values for pigs held under long day conditions (LD) are shown in red, values for pigs held under short day conditions (SD) are shown in blue. The graphs and statistical analyses include values calculated from the 26 samplings per pig, subdivided into values during lights-on or lights-off for all animals per treatment (mean ± SEM, LD *n* = 9, lights-on = 18 samplings & lights-off = 8 samplings, SD *n* = 11, lights-on = 10 samplings & lights-off = 16 samplings). Mixed ANOVA with treatment (LD/SD) as between-subjects factor and light (on/off) as within-subjects repeated factor was performed for comparisons. If there was a significant interaction effect, simple main effects were analyzed with the least significant difference (LSD) procedure. Significant interactions between main effects were found for **(A)** plasma cortisol concentration and **(B)** activity behavior, whereas for **(C)** hematocrit, there was a significant main effect of light only; refer to Table S9 for statistical details, ^***^P < 0.001, ^*^P < 0.05.

Potential associations of the experimental factors treatment, light, and concentrate feeding as well as the covariates plasma cortisol concentration, activity behavior, hematocrit, and sampling with the cell numbers of the various leukocyte types in porcine blood were assessed with generalized linear mixed models (Table 2). Concerning the factors treatment and light, mixed model results resembled the findings of cosinor analyses. In accordance with the absent differences in mesor or mean values, we also found no association of immune cell numbers in porcine blood with LD or SD treatment. Cell numbers of immune cell types, which peaked in the dark phase or in the late light phase in porcine blood according to cosinor analyses, were found to be positively associated with lights-off, whereas NK cell and neutrophil counts, peaking during the early and mid-light phase, were found to be negatively associated with lights-off. Porcine monocyte and Ag-exp. Th cell counts were not associated with the factor light. No association of concentrate feeding with immune cell numbers in porcine blood was found for the major proportion of immune cell types, except for total leukocyte, NK cell, monocyte, neutrophil, and eosinophil counts, which were negatively associated with concentrate feeding. Whereas cell numbers in porcine blood for the most immune cell types were negatively associated with plasma cortisol concentration, positive associations were found for NK cell and Ag-exp. Th cell counts and no association was found for neutrophil counts. Furthermore, activity behavior was negatively associated with cell numbers in blood in almost all investigated immune cell types, except for NK cells and neutrophils, for which positive associations were found. As expected, hematocrit was positively associated with the cell numbers in blood of all investigated immune cell types. Whereas the major proportion of investigated immune cell types showed no association between cell numbers in porcine blood and repeated sampling, a minor proportion was negatively associated with sampling. In addition, evaluation of experimental effects on the investigated covariates with mixed ANOVA revealed interaction effects of treatment and light for plasma cortisol concentration and activity behavior, whereas for hematocrit, there was a main effect of light only (Figure 6).

**Table 2:** Potential associations of various fixed effects with immune cell numbers in porcine blood^a^.

**Variable**	**Fixed effects: Estimated associations^b^ with significance levels**^c^					
	**Treatment (LD)**	**Light (off)**	**Feeding (yes)**	**Cortisol**	**Activity**	**Hematocrit**	**Sampling**
Leukocytes	↔	↑***	↓***	↓***	↓***	↑***	↔
NK cells	↔	↓***	↓*	↑***	↑***	↑***	↓***
T cells	↔	↑***	↔	↓***	↓***	↑***	↔
Monocytes	↔	↔	↓***	↓***	↓**	↑***	↓**
Neutrophils	↔	↓***	↓***	↔	↑**	↑***	↓*
Eosinophils	↔	↑***	↓***	↓***	↓***	↑***	↔
Total Th cells	↔	↑***	↔	↓***	↓***	↑***	↓*
Cytotoxic T cells	↔	↑***	↔	↓**	↓***	↑**	↔
γδ T cells	↔	↑***	↔	↓***	↓***	↑***	↔
Naive Th cells	↔	↑***	↔	↓***	↓***	↑***	↔
Ag-exp. Th cells	↔	↔	↔	↑*	↓**	↑***	↓**

a *Potential associations were assessed by generalized linear mixed model analyses: y_ij_ = μ + treatment_i_ + light_ij_ + concentrate feeding_j_ + plasma cortisol concentration_ij_ + relative activity behavior_ij_ + hematocrit_ij_ + sampling_j_ + animal identity_i_ + ε_ij_. Thereby, y_ij_ represents the cell number/μl blood of the specific immune cell population in porcine blood for an animal i at sampling j, the intercept μ, the factors treatment (LD/SD), light (off/on), and concentrate feeding (yes/no) as well as the covariates plasma cortisol concentration (ng/ml), relative activity behavior (%), hematocrit (%), and sampling (1–26) were set as fixed effects. The factor animal identity (n = 20) was set as random effect with a scaled identity (ID) covariance structure. Sampling was set as repeated effect with a first order autoregressive (AR(1)) residual covariance structure (ε_ij_). Animal identity was set as subject. Detailed model results can be found in Table S2*.

b Estimated association:

↑ *positive*,

↓ *negative*,

↔ *none*.

^c^

*** *P < 0.001*,

** *P < 0.01*,

* *P < 0.05*.

## Discussion

The present study demonstrated that the photoperiod modulates diurnal rhythms in cell numbers of different immune cell types in blood of domestic pigs. Distinct photoperiodic differences were found in relative amplitudes and peak times but not in mesor or mean values of cell numbers in certain immune cell types in porcine blood. To our knowledge, this is the first study investigating the effect of different photoperiods on diurnal rhythms in peripheral immune cell numbers within the same season in any species. We found diurnal rhythms in immune cell numbers in porcine blood under both lighting regimes for all investigated immune cell types except for Ag-exp. Th cells, which is consistent with our previous results for domestic pigs held under 12L:12D lighting conditions (39).

Interestingly, on individual level, higher proportions of animals held under SD than LD exhibited diurnal rhythms in cell numbers in several of the investigated leukocyte types in blood. In addition, several immune cell types oscillated stronger under SD than LD in the present study. This finding is in consistence with previous reports showing marked diurnal rhythmicity in leukocyte counts during winter months, i.e., under SD, and a weakening or loss of rhythmicity in summer months, i.e., under LD, in humans and nocturnal rodents (31, 33, 34). The present study also found a potentially greater dispersal of peak times under LD in most cell types. Thus, our results imply stronger rhythmicity in leukocyte counts in general under SD and weakened rhythmicity under LD. It was previously shown in mice that exposure to LD leads to weakened intercellular coupling within the SCN compared to SD, which is associated with greater dispersal of peak times in clock gene expression within the anterior SCN under LD (51). Further investigations should attempt to clarify if this mechanism is also present in domestic pigs and if it is directly linked to weakened rhythmicity and dispersed peak times in immune cell counts seen under LD.

Several leukocyte types peaked earlier relative to the time of lights-on under SD than LD. The consistency in effect direction for most investigated immune cell types displaying photoperiodic differences in relative amplitudes and peak times in cell numbers in porcine blood suggests that the experimental photoperiod acted on those immune cell types through presumably common intrinsic mechanisms. Moreover, it can be assumed that light or darkness (53) likely acted as main stimulus, called zeitgeber, on diurnal rhythmicity in those cell types. Notably, it is not known whether the time of lights-on or lights-off, mid-day, mid-night, the relative span of light or darkness per day, or a combination of these phenomena is most important for mediating photoperiodic effects on diurnal immune rhythms. The photoperiodic effect was particularly pronounced in total leukocytes, NK cells, and T cells including naive Th cells in the present study, which is in consistence with the above mentioned studies (31, 33, 34). In previous studies, it was shown that plasma cortisol and the rest-activity cycle represent main driving forces of diurnal rhythms in immune cell numbers in blood (37, 54). The results of generalized linear mixed model analyses of the present study support this concept, as those immune cell types, which exhibited marked differences in relative amplitudes and peak times in cell numbers in porcine blood, were associated with plasma cortisol concentration and activity behavior, resembling previous results (55–58). Monocyte counts did not differ in peak times but oscillated stronger under SD in blood of domestic pigs. As only half of the pigs held under LD but two-thirds of pigs held under 12L:12D in our previous study (39) and all pigs held under SD exhibited diurnal rhythms in monocyte numbers in blood, photoperiod seems to be essential for diurnal rhythmicity in this cell type as well. Few investigated leukocyte types neither displayed differences in relative amplitudes nor in peak times of immune cell counts. Interestingly, diurnal rhythms in neutrophil numbers in porcine blood seem to be regulated differently from other immune cell types. Neutrophil counts were found not to be associated with plasma cortisol concentration, which is supported by other studies (39, 49, 55). As animals under both lighting regimes were fed simultaneously, feeding time potentially displays an important zeitgeber for this cell type (59). This hypothesis is supported by mixed model results revealing them as the only investigated cell type being associated with feeding while lacking association with plasma cortisol concentration. Notably, the association with feeding potentially involves activity behavior as mediating factor (57). Alternatively, the time of lights-on might be more important for diurnal rhythms in neutrophil counts than the photoperiod. The non-significant results for total Th cells might have been influenced by the proportion of Ag-exp. Th cells, which lack overall diurnal rhythmicity in the present study and under 12L:12D lighting conditions as well (39).

The present study also demonstrated photoperiodic effects on diurnal rhythms in plasma cortisol concentrations and activity behavior. Both variables exhibited earlier peak times relative to the time of lights-on under SD, emphasizing the importance of light or darkness as zeitgeber (53, 60). Our results for plasma cortisol concentration and activity behavior are in remarkable agreement with human and primate studies (61–63). Interestingly, the interaction effect of treatment with light in activity behavior seemed to result from the observed stronger diurnal oscillation under SD than LD. Thereby, pigs held under SD were more active during lights-on than animals held under LD, probably compensating for the shorter time span of lights-on. This effect was already described for pigs as well as in primate studies (63, 64).

In contrast to photoperiodic effects in peak times and relative amplitudes, no differences in mesor or mean values of cell numbers in porcine blood of any investigated leukocyte type were found in the present study. This result is in contrast to previous reports demonstrating seasonal or photoperiodic differences in immune cell counts in mammals, with generally greater cell numbers in autumn, winter, or under SD (24, 26, 27, 31–34, 36, 65–68). There may be several possible reasons to explain this discrepancy. Some of the above mentioned studies sampled each subject or animal at a single point in time within 24 h to compare different photoperiods or seasons (24, 26, 27, 65–68). It was pointed out by some authors that such single point measurements should be interpreted with caution as observed differences might not result from a change in mesor but rather from a shift in peak time or change in strength of diurnal oscillation (or both) between lighting regimes or seasons (69, 70). The experimental design used in the present study meets the recommendation of at least 12 time points per cycle across 2 full cycles for studying biological rhythms (71). Studies analyzing diurnal rhythms during different seasons showed differences in mesor values in leukocyte counts (31–34, 36). However, these studies evaluated the effect of season and not photoperiod in particular and investigated fewer time points across just 1 diurnal cycle. Thus, whether the differences in experimental settings between the above mentioned studies and the present study led to the observed discrepancy in mesor values cannot be answered yet. The crossbred domestic pigs used in the present study can enhance comparability to human studies compared to inbred models. Thereby, they reveal a high variability in total immune cell numbers between individuals, whereas the course of diurnal immune rhythms is highly comparable [refer to (39)]. Although photoperiodic differences were previously found with smaller numbers of hamsters (67), this inter-individual variability in pigs could have impeded the identification of significant effects in mesor or mean values. Further investigations using intra-individual approaches could elucidate this issue. Additionally, the above mentioned effect might have been enhanced by the use of castrated male pigs in the present study. It was suggested that gonadal hormones can play a modifying role in photoperiod-induced immune modulations, at least in seasonal breeding species (20, 21, 23, 72). Further investigations with gonad-intact domestic pigs could elucidate, whether gonadal hormones influence total leukocyte counts in this moderately seasonal breeding species (73). Apart from this, few studies investigating diurnal rhythms in rodents held under 12L:12D lighting conditions throughout the whole year found differences in mesor values of immune cell numbers (36, 70). These results in conjunction with the lacking photoperiodic effect on mesor values of leukocyte counts in the present study suggest that the proposed central circannual clock (8–10) and not the photoperiod might influence mesor values of leukocyte numbers in the pig.

Plasma cortisol concentration and activity behavior also did not exhibit photoperiodic differences in mesor or mean values in the present study. Whereas, several studies investigating plasma cortisol concentration throughout the year in humans found higher diurnal mesor or mean values during the winter season (74–76), results of other human studies were inconsistent (77, 78). Remarkably, comparing our results with a primate study using the same experimental setting as we did reveals striking comparability in mesor or mean values of cortisol and activity behavior (63). Hematocrit was the only variable exhibiting a photoperiodic difference in mesor values, although mean values were not different and other studies also did not find seasonal or photoperiodic effects on hematocrit (24, 67, 79, 80).

In conclusion, the present study investigated, to our knowledge, for the first time in any species photoperiodic effects on diurnal rhythms in immune cell numbers in blood. Distinct photoperiodic differences in relative amplitudes and peak times in the cell numbers of certain leukocyte types in blood were found in domestic pigs, whereas there was no difference in mesor or mean values. Our results imply stronger rhythmicity in leukocyte counts in general under SD. Moreover, common intrinsic mechanisms seem to regulate photoperiodic effects on diurnal rhythms in cell counts of most porcine leukocyte types, except for neutrophils. Whether these differences influence disease susceptibility of domestic pigs cannot be answered yet. Human and primate studies using experimental settings similar to the present study, found comparable results for plasma cortisol concentration and activity behavior (61, 63), strengthening the importance of the domestic pig as suitable large animal model. Therefore, the domestic pig provides the opportunity to further elucidate the influence of environmental factors on diurnal and seasonal immune rhythms as well as the importance of these rhythms for immune competence and disease susceptibility in diurnally active species.

## Data Availability

All datasets generated for this study are included in the manuscript and/or the supplementary files.

## Author Contributions

SS conceived and designed the study. LE and SS designed experiments, performed research, analyzed and interpreted the data, and wrote the manuscript. UW conducted the cortisol analyses and supervised surgery. BP conducted the behavioral analyses. VS assisted in experimental design and manuscript preparation. All authors read and approved the submitted version of the manuscript.

### Conflict of Interest Statement

The authors declare that the research was conducted in the absence of any commercial or financial relationships that could be construed as a potential conflict of interest.
