# Supplementary material for: Photoperiodic Effects on Diurnal Rhythms in Cell Numbers of Peripheral Leukocytes in Domestic Pigs
Source: Front Immunol. 2019 Mar 12;10:393. doi: 10.3389/fimmu.2019.00393 (PMC6422931; doi:10.3389/fimmu.2019.00393)
Supplement: Supplementary file 1 [file Data_Sheet_1.PDF]

Supplementary Material

Photoperiodic Effects on Diurnal Rhythms in Cell Numbers of Peripheral Leukocytes in Domestic Pigs

Larissa C. Engert, Ulrike Weiler, Birgit Pfaffinger, Volker Stefanski, Sonja S. Schmucker*

Behavioral Physiology of Livestock, Institute of Animal Science, University of Hohenheim, Stuttgart, Germany

*** Correspondence:**
Dr. Sonja S. Schmucker
Sonja.Schmucker@uni-hohenheim.de

Contents

FIGURE S1 | Mean values 2
FIGURE S2 | Cell type comparisons of relative amplitudes 3
FIGURE S3 | Cell type comparisons of peak times 4
FIGURE S4 | Diurnal rhythms of the cell numbers of CD8α^{-} and CD8α^{+} γδ T cells..........5
FIGURE S5 | Diurnal profiles (mean ± SEM) of the investigated variables in the study..........6
TABLE S1 | Results of individual cosinor analyses per animal..........7
TABLE S2 | Results of generalized linear mixed model analyses 8
TABLE S3 | Results of overall cosinor analyses for CD8α^{-} and CD8α^{+} γδ T cells..........11
TABLE S4 | Summary of generalized linear mixed models for CD8α^{-} and CD8α^{+} γδ T cells 11
TABLE S5 | Statistical details for pairwise comparisons of mesor values 12
TABLE S6 | Statistical details for pairwise comparisons of relative amplitudes..........13
TABLE S7 | Statistical details for pairwise comparisons of peak times 14
TABLE S8 | Statistical details for pairwise comparisons of mean values 15
TABLE S9 | Statistical details for mixed ANOVA 16

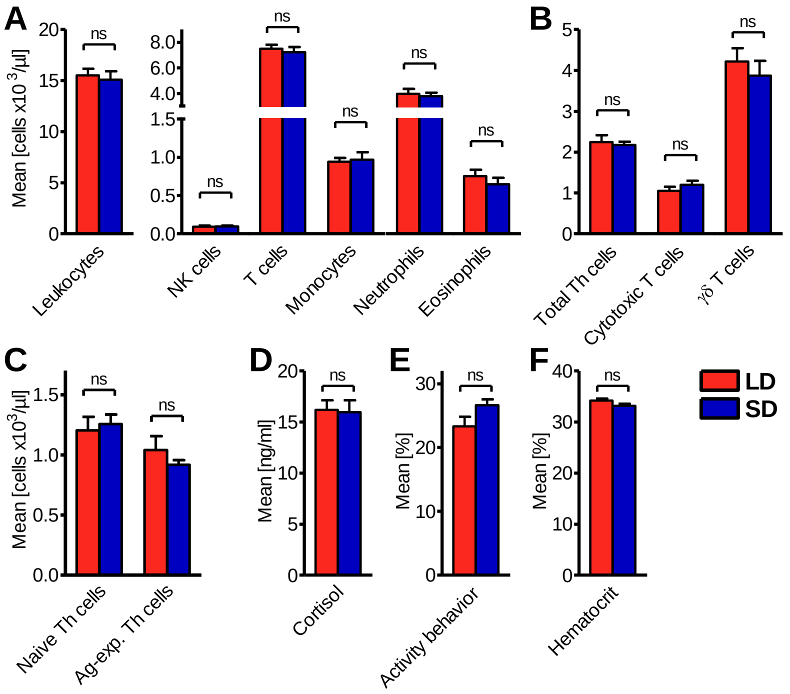

FIGURE S1 | Mean values of the cell numbers of different immune cell populations in blood, plasma cortisol concentration, activity behavior, and hematocrit in domestic pigs. Values for pigs held under long day conditions (LD) are shown in red, values for pigs held under short day conditions (SD) are shown in blue for **(A)** main immune cell populations, **(B)** different T cell subpopulations, **(C)** Th cell subtypes with distinctive differentiation states, **(D)** plasma cortisol concentration, **(E)** activity behavior, and **(F)** hematocrit. The graphs and statistical analyses include values calculated from the 26 samplings per pig for all animals per treatment (mean ± SEM, LD n = 9, SD n = 11). Pairwise comparisons were performed with two-tailed, unpaired Student's *t*-tests (total leukocytes, T cells, monocytes, neutrophils, eosinophils, γδ T cells, plasma cortisol concentration, and hematocrit), unequal variance *t*-test (activity behavior), or two-tailed Mann-Whitney *U* tests (NK cells, total Th cells, cytotoxic T cells, naive Th cells, and Ag-exp. Th cells); refer to Table S8 in Supplementary Material for statistical details, ns $P \geq 0.05$.

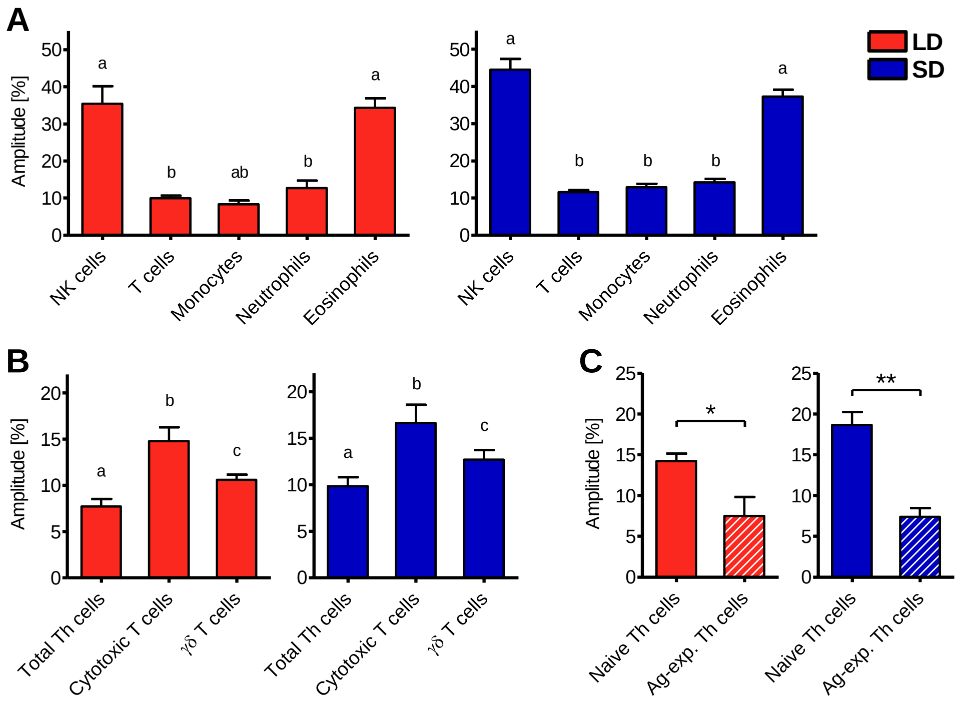

FIGURE S2 | Cell type comparisons of relative amplitudes of the cell numbers of different immune cell populations in porcine blood within treatments. Values for pigs held under long day conditions (LD) are shown in red, values for pigs held under short day conditions (SD) are shown in blue. The graphs and statistical analyses only include values of animals with significant ($P < 0.05$) diurnal rhythm in individual cosinor analyses in all compared cell types, respectively (mean ± SEM, LD n = 5–8, SD n = 7–11, refer to Table S1 in Supplementary Material). Hatched bars indicate missing diurnal rhythm in overall cosinor analyses with combined datasets of all animals per treatment (significant diurnal rhythm at $P < 0.05$, refer to Table 1 in main text). **(A)** Multiple comparisons within main immune cell populations for pigs held under LD (n = 5) were performed with Friedman test ($\chi^2(4) = 16.48$, $P = 0.002$; NK cells: 25th–75th percentile (Pctl) 27.5%–45.1%, T cells: Pctl 8.3%–11.2%, monocytes: Pctl 6.3%–10.4%, neutrophils: Pctl 9.7%–16.7%, eosinophils: Pctl 29.6%–39.7%) followed by Wilcoxon signed-rank *post hoc* tests with Benjamini-Hochberg correction and for pigs held under SD (n = 10) with repeated measures ANOVA with Greenhouse-Geisser correction ($F(1.99,17.92) = 85.21$, $P = 7.16 \times 10^{-10}$; NK cells: 95% confidence interval (CI) 37.9%–51.0%, T cells: CI 10.3%–12.8%, monocytes: CI 10.7%–15.1%, neutrophils: CI 12.1%–16.3%, eosinophils: CI 33.0%–41.5%) followed by Bonferroni *post hoc* tests. **(B)** Multiple comparisons within different T cell subpopulations for pigs held under LD (n = 8) were performed with repeated measures ANOVA ($F(2,14) = 19.51$, $P = 8.96 \times 10^{-05}$; total Th cells: CI 5.8%–9.6%, cytotoxic T cells: CI 11.2%–18.3%, γδ T cells: CI 9.3%–11.9%) followed by Bonferroni *post hoc* tests and for pigs held under SD (n = 11) with Friedman test ($\chi^2(2) = 9.46$, $P = 0.009$; total Th cells: Pctl 7.0%–12.1%, cytotoxic T cells: Pctl 10.4%–19.0%, γδ T cells: Pctl 10.8%–13.3%) followed by Wilcoxon signed-rank *post hoc* tests with Bonferroni-Holm correction. Different letters indicate significant differences at $P < 0.05$. **(C)** Pairwise comparisons between Th cell subtypes with distinctive differentiation states (LD n = 6, SD n = 7) were performed with two-tailed, paired Student's *t*-tests (LD: $t(5) = 2.66$, $P = 0.045$; naive Th cells: CI 11.9%–16.6%, Ag-exp. Th cells: CI 1.4%–13.6%; SD: $t(6) = 5.27$, $P = 0.002$; naive Th cells: CI 14.8%–22.5%, Ag-exp. Th cells: CI 4.8%–10.0%); ** $P < 0.01$, * $P < 0.05$.

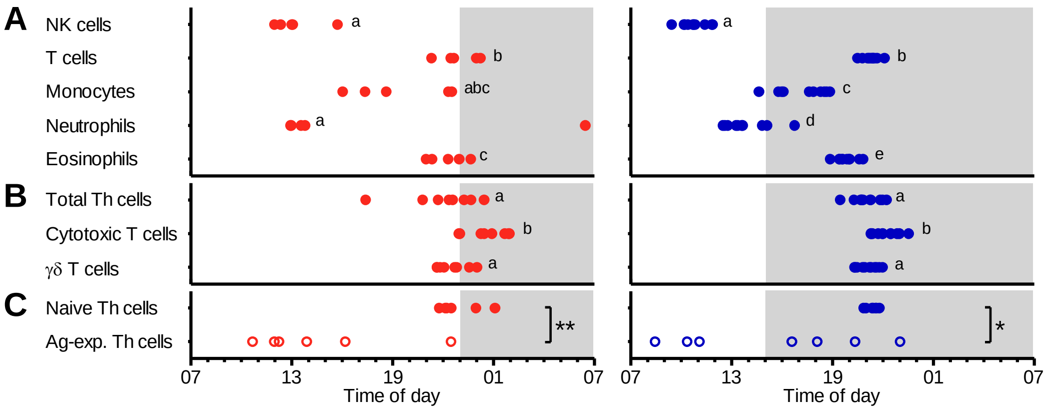

FIGURE S3 | Cell type comparisons of peak times of the cell numbers of different immune cell populations in porcine blood within treatments. Values for pigs held under long day conditions (LD) are shown in red, values for pigs held under short day conditions (SD) are shown in blue. Shaded areas indicate lights-off in the respective treatments. The graphs and statistical analyses only include values of animals with significant ($P < 0.05$) diurnal rhythm in individual cosinor analyses in all compared cell types, respectively (LD n = 5–8, SD n = 7–11, refer to Table S1 in Supplementary Material). Circles indicate missing diurnal rhythm in overall cosinor analyses with combined datasets of all animals per treatment (significant diurnal rhythm at $P < 0.05$, refer to Table 1 in main text). **(A)** Multiple comparisons within main immune cell populations for pigs held under LD (n = 5) were performed with Friedman test ($\chi^2(4) = 17.12$, $P = 0.002$; NK cells: 25th–75th percentile (Pctl) 12:10–14:24, T cells: Pctl 21:53–00:07, monocytes: Pctl 16:42–22:26, neutrophils: Pctl 09:42–13:41, eosinophils: Pctl 21:11–23:19) followed by Wilcoxon signed-rank *post hoc* tests with Benjamini-Hochberg correction and for pigs held under SD (n = 10) with repeated measures ANOVA ($F(4,36) = 302.35$, $P = 3.67 \times 10^{-27}$; NK cells: 95% confidence interval (CI) 10:13–11:19, T cells: CI 20:58–21:37, monocytes: CI 16:10–18:18, neutrophils: CI 12:53–14:48, eosinophils: CI 19:24–20:14) followed by Bonferroni *post hoc* tests. **(B)** Multiple comparisons within different T cell subpopulations for pigs held under LD (n = 8) and SD (n = 11) were performed with repeated measures ANOVA (LD: $F(2,14) = 10.92$, $P = 0.001$; total Th cells: CI 20:12–23:52, cytotoxic T cells: CI 23:38–01:39, γδ T cells: CI 21:47–23:18; SD: $F(2,20) = 10.67$, $P = 7.03 \times 10^{-04}$; total Th cells: CI 20:29–21:34, cytotoxic T cells: CI 21:41–22:41, γδ T cells: CI 20:43–21:31) followed by Bonferroni *post hoc* tests. Different letters indicate significant differences at $P < 0.05$. **(C)** Pairwise comparisons between Th cell subtypes with distinctive differentiation states (LD n = 6, SD n = 7) were performed with two-tailed, paired Student's *t*-tests (LD: $t(5) = 5.99$, $P = 0.002$; naive Th cells: CI 21:35–00:18, Ag-exp. Th cells: CI 10:03–19:05; SD: $t(6) = 2.94$, $P = 0.026$; naive Th cells: CI 21:04–21:41, Ag-exp. Th cells: CI 10:18–20:32); ** $P < 0.01$, * $P < 0.05$.

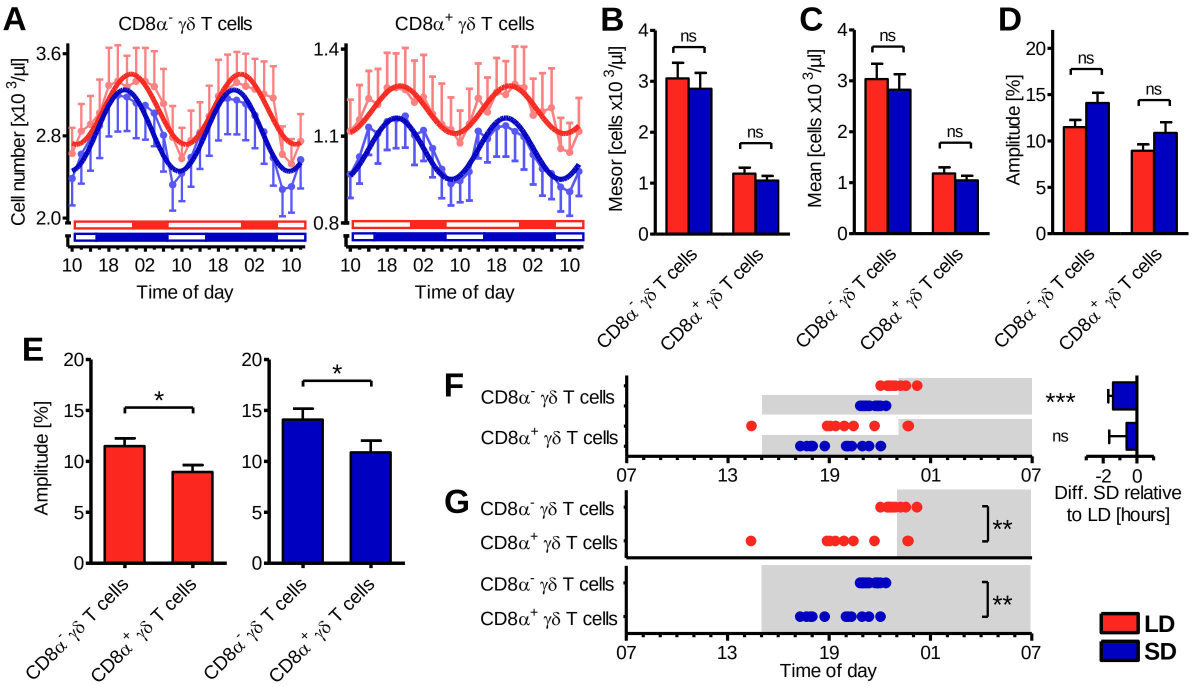

FIGURE S4 | Diurnal rhythms of the cell numbers of CD8α$^-$ and CD8α$^+$ γδ T cells in porcine blood. Values for pigs held under long day conditions (LD) are shown in red, values for pigs held under short day conditions (SD) are shown in blue. Shaded areas indicate lights-off in the respective treatments. **(A)** Open bars indicate light periods, filled bars indicate dark periods for the respective lighting regime. Measured cell numbers in porcine blood are shown in pale color per treatment (mean ± SEM, LD n = 9, SD n = 11). Dark curves correspond to the results of overall cosinor analyses with combined datasets of all animals per treatment (LD n = 9, SD n = 11, significant diurnal rhythm at $P < 0.05$, refer to Table S3 in Supplementary Material). **(B)** Mesor values calculated from individual cosinor analyses of all animals per treatment (all animals displayed significant diurnal rhythm at $P < 0.05$, refer to Table S1 in Supplementary Material), **(C)** mean values calculated from the 26 samplings per pig for all animals per treatment, and **(D)** relative amplitudes calculated from individual cosinor analyses of all animals per treatment were compared with two-tailed, unpaired Student's *t*-tests (mean ± SEM, LD n = 9, SD n = 11). **(E)** Cell type comparisons of relative amplitudes within treatments were performed with two-tailed, paired Student's *t*-tests (mean ± SEM, LD n = 9, SD n = 11; LD: $t(8) = 2.89$, $P = 0.020$; CD8α$^-$ γδ T cells: 95% confidence interval (CI) 9.8%–13.3%, CD8α$^+$ γδ T cells: CI 7.4%–10.5%; SD: $t(10) = 2.71$, $P = 0.022$; CD8α$^-$ γδ T cells: CI 11.7%–16.5%, CD8α$^+$ γδ T cells: CI 8.3%–13.5%). **(F)** Peak times calculated from individual cosinor analyses of all animals per treatment were compared with two-tailed, unpaired Student's *t*-tests (LD n = 9, SD n = 11). The bar graph on the right side illustrates the differences in peak times of SD relative to LD (mean ± SEM). (**G**) Cell type comparisons of peak times within treatments were performed with two-tailed, paired Student's *t*-tests (LD: $t(8) = 3.36$, $P = 0.010$; CD8α$^-$ γδ T cells: CI 22:26–23:26, CD8α$^+$ γδ T cells: CI 17:57–22:18; SD: $t(10) = 4.40$, $P = 0.001$; CD8α$^-$ γδ T cells: CI 21:10–21:51, CD8α$^+$ γδ T cells: CI 18:24–20:36); refer to Tables S5–S8 in Supplementary Material for statistical details, *** $P < 0.001$, ** $P < 0.01$, * $P < 0.05$, ns $P \geq 0.05$.

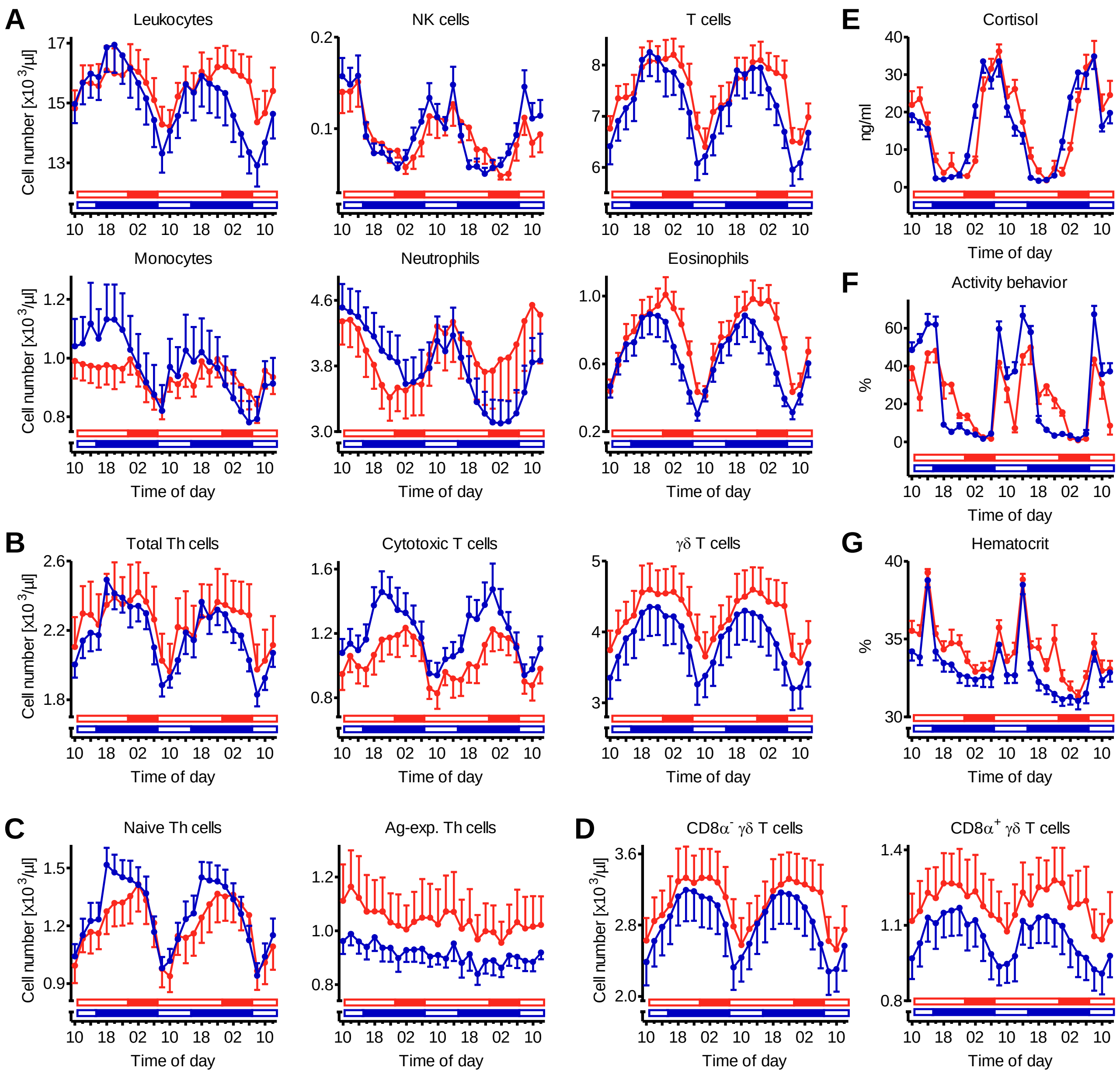

FIGURE S5 | Diurnal profiles (mean ± SEM) of the investigated variables in the study. Values for pigs held under long day conditions (LD n = 9) are shown in red, values for pigs held under short day conditions (SD n = 11) are shown in blue. For visual evaluation purposes, the figure illustrates the same datasets as Figure 2 in main text and Figure S4A in Supplementary Material just without the plotted cosine curves. Diurnal profiles in cell numbers in porcine blood are depicted for **(A)** main immune cell populations, **(B)** different T cell subpopulations, **(C)** Th cell subtypes with distinctive differentiation states, and **(D)** CD8α^{-} and CD8α^{+} γδ T cells. In addition, diurnal profiles in **(E)** plasma cortisol concentration, **(F)** activity behavior (values represent the proportion of time, in which the animals were active within the two-hour interval preceding blood sampling), and **(G)** hematocrit are shown.

TABLE S1 | Results of individual cosinor analyses per animal

Variable	Treatment	n[a]	Prop.[b]	Mesor	Amplitude	Amplitude [%][c]	Peak time[d]
Leukocytes [/µl]	LD	7	78%	16214.6 ± 615.3	979.7 ± 60.4	6.0 ± 0.3	21:43 ± 00:52
	SD	11	100%	15156.1 ± 819.3	1380.5 ± 178.1	8.9 ± 0.7	19:02 ± 00:24
NK cells [/µl]	LD	9	100%	91.5 ± 14.0	32.5 ± 7.0	33.6 ± 3.3	13:10 ± 00:23
	SD	11	100%	93.1 ± 11.3	41.8 ± 5.7	44.2 ± 2.6	10:50 ± 00:14
T cells [/µl]	LD	9	100%	7548.4 ± 308.1	698.6 ± 73.8	9.2 ± 0.9	22:46 ± 00:19
	SD	11	100%	7283.3 ± 406.1	920.2 ± 111.9	12.5 ± 1.1	21:22 ± 00:09
Monocytes [/µl]	LD	5	56%	915.5 ± 45.4	76.0 ± 8.1	8.3 ± 1.0	19:23 ± 01:19
	SD	11	100%	968.0 ± 99.1	126.9 ± 19.7	12.9 ± 0.9	17:28 ± 00:29
Neutrophils [/µl]	LD	9	100%	3946.9 ± 379.6	516.1 ± 88.4	12.7 ± 1.2	12:15 ± 00:45
	SD	10	91%	3592.5 ± 248.4	509.9 ± 51.1	14.2 ± 0.9	13:51 ± 00:25
Eosinophils [/µl]	LD	9	100%	770.9 ± 82.9	247.2 ± 23.1	32.8 ± 1.7	22:03 ± 00:17
	SD	11	100%	658.7 ± 86.5	252.9 ± 37.1	37.8 ± 1.8	19:51 ± 00:10
Total Th cells [/µl]	LD	8	89%	2318.1 ± 179.1	176.8 ± 21.3	7.7 ± 0.8	22:02 ± 00:46
	SD	11	100%	2187.3 ± 78.7	217.5 ± 23.3	9.8 ± 1.0	21:02 ± 00:15
Cytotoxic T cells [/µl]	LD	9	100%	1055.3 ± 102.5	149.6 ± 20.6	14.0 ± 1.5	00:42 ± 00:23
	SD	11	100%	1210.3 ± 101.0	211.2 ± 40.5	16.7 ± 2.0	22:11 ± 00:13
Total γδ T cells [/µl]	LD	9	100%	4249.4 ± 328.3	423.9 ± 40.6	10.1 ± 0.7	22:26 ± 00:18
	SD	11	100%	3908.1 ± 362.1	502.0 ± 69.5	12.7 ± 1.0	21:07 ± 00:11
Naive Th cells [/µl]	LD	9	100%	1216.3 ± 115.5	179.0 ± 21.9	14.5 ± 0.8	23:04 ± 00:25
	SD	11	100%	1271.9 ± 79.9	222.4 ± 15.8	17.7 ± 1.1	21:23 ± 00:06
Ag-exp. Th cells [/µl]	LD	6	67%	1107.6 ± 165.6	82.9 ± 27.8	7.5 ± 2.4	14:34 ± 01:45
	SD	7	64%	921.1 ± 56.0	67.5 ± 9.4	7.4 ± 1.1	15:25 ± 02:05
$CD8\alpha^{-}$ γδ T cells [/µl]	LD	9	100%	3058.1 ± 305.3	345.4 ± 34.3	11.5 ± 0.8	22:56 ± 00:13
	SD	11	100%	2851.3 ± 311.0	399.5 ± 53.7	14.1 ± 1.1	21:30 ± 00:09
$CD8\alpha^{+}$ γδ T cells [/µl]	LD	9	100%	1191.2 ± 120.6	104.5 ± 12.3	9.0 ± 0.7	20:07 ± 00:57
	SD	11	100%	1056.8 ± 92.5	114.7 ± 16.7	10.9 ± 1.2	19:30 ± 00:30
Cortisol [ng/ml]	LD	9	100%	15.4 ± 0.9	14.9 ± 1.2	97.6 ± 6.2	08:19 ± 00:15
	SD	11	100%	15.5 ± 1.1	15.6 ± 1.3	100.0 ± 3.4	06:34 ± 00:12
Activity behavior [%]	LD	9	100%	22.7 ± 1.4	16.6 ± 2.0	71.3 ± 5.1	15:12 ± 00:21
	SD	11	100%	24.7 ± 0.8	28.7 ± 1.3	116.1 ± 2.9	12:33 ± 00:11
Hematocrit [%]	LD	8	89%	34.4 ± 0.4	1.7 ± 0.1	5.1 ± 0.5	14:51 ± 00:17
	SD	11	100%	33.1 ± 0.4	1.7 ± 0.1	5.0 ± 0.5	13:36 ± 00:15

Values are presented as mean ± SEM.
[a] Number of animals out of 9 under long day conditions (LD) or out of 11 under short day conditions (SD) with significant ($P < 0.05$) diurnal rhythm in individual cosinor analyses
[b] Proportion of animals displaying diurnal rhythmicity
[c] Relative amplitude (amplitude/mesor)
[d] Time of day ± hh:mm

TABLE S2 | Results of generalized linear mixed model analyses[a]

Variable	Model term	Fixed coefficient ± SE	df [b]	F	P
Leukocytes[c]	*Corrected model* (Coefficient: Intercept)	9.2278 ± 0.0646	7,512.00	31.86	$<1.00 \times 10^{-36}$
	Treatment (LD)	0.0227 ± 0.0680	1,18.01	0.11	0.742
	Light (off)	0.0228 ± 0.0065	1,492.94	12.25	5.07×10^{-04}
	Feeding (yes)	-0.0233 ± 0.0066	1,445.61	12.34	4.89×10^{-04}
	Cortisol (per 1 ng/ml)	-0.0009 ± 0.0002	1,482.21	20.61	7.13×10^{-06}
	Activity (per 1%)	-0.0008 ± 0.0001	1,415.21	71.21	4.44×10^{-16}
	Hematocrit (per 1%)	0.0127 ± 0.0013	1,426.69	97.29	$<1.00 \times 10^{-36}$
	Sampling (per sample)	-0.0011 ± 0.0008	1,42.67	1.79	0.189
NK cells[c]	*Corrected model* (Coefficient: Intercept)	2.8140 ± 0.2831	7,512.00	82.19	$<1.00 \times 10^{-36}$
	Treatment (LD)	-0.1151 ± 0.1808	1,18.07	0.41	0.532
	Light (off)	-0.2433 ± 0.0313	1,399.94	60.57	6.13×10^{-14}
	Feeding (yes)	-0.0915 ± 0.0379	1,424.12	5.82	0.016
	Cortisol (per 1 ng/ml)	0.0083 ± 0.0010	1,339.90	75.02	2.22×10^{-16}
	Activity (per 1%)	0.0031 ± 0.0006	1,424.25	30.34	6.29×10^{-08}
	Hematocrit (per 1%)	0.0500 ± 0.0073	1,478.84	46.91	2.28×10^{-11}
	Sampling (per sample)	-0.0077 ± 0.0020	1,114.16	14.38	2.41×10^{-04}
T cells[c]	*Corrected model* (Coefficient: Intercept)	8.4669 ± 0.0768	7,512.00	51.15	$<1.00 \times 10^{-36}$
	Treatment (LD)	0.0485 ± 0.0711	1,18.05	0.46	0.504
	Light (off)	0.0712 ± 0.0084	1,490.35	71.68	2.22×10^{-16}
	Feeding (yes)	-0.0038 ± 0.0088	1,435.32	0.19	0.664
	Cortisol (per 1 ng/ml)	-0.0012 ± 0.0003	1,494.34	21.04	5.70×10^{-06}
	Activity (per 1%)	-0.0015 ± 0.0001	1,396.37	129.49	$<1.00 \times 10^{-36}$
	Hematocrit (per 1%)	0.0127 ± 0.0017	1,417.97	55.35	5.78×10^{-13}
	Sampling (per sample)	-0.0008 ± 0.0008	1,48.80	0.95	0.334
Monocytes[c]	*Corrected model* (Coefficient: Intercept)	6.2428 ± 0.1308	7,512.00	19.26	$<1.00 \times 10^{-36}$
	Treatment (LD)	0.0054 ± 0.1426	1,18.00	<0.01	0.970
	Light (off)	-0.0160 ± 0.0123	1,484.10	1.69	0.194
	Feeding (yes)	-0.0787 ± 0.0130	1,443.07	36.69	2.96×10^{-09}
	Cortisol (per 1 ng/ml)	-0.0018 ± 0.0004	1,491.91	21.83	3.84×10^{-06}
	Activity (per 1%)	-0.0006 ± 0.0002	1,409.84	9.54	0.002
	Hematocrit (per 1%)	0.0205 ± 0.0025	1,432.09	65.06	7.33×10^{-15}
	Sampling (per sample)	-0.0038 ± 0.0011	1,66.93	11.53	0.001
Neutrophils[d]	*Corrected model* (Coefficient: Intercept)	7.8673 ± 0.1102	7,512.00	13.63	3.33×10^{-16}
	Treatment (LD)	0.0461 ± 0.1197	1,17.89	0.15	0.705
	Light (off)	-0.0536 ± 0.0106	1,480.00	25.36	6.75×10^{-07}
	Feeding (yes)	-0.0687 ± 0.0109	1,460.62	39.54	7.49×10^{-10}
	Cortisol (per 1 ng/ml)	-0.0005 ± 0.0003	1,465.15	2.65	0.104
	Activity (per 1%)	0.0004 ± 0.0002	1,439.91	7.37	0.007
	Hematocrit (per 1%)	0.0133 ± 0.0021	1,452.83	40.63	4.53×10^{-10}
	Sampling (per sample)	-0.0041 ± 0.0020	1,28.65	4.30	0.047
Eosinophils[e]	*Corrected model* (Coefficient: Intercept)	-50.46 ± 105.91	7,71.39	20.40	8.99×10^{-15}
	Treatment (LD)	64.65 ± 82.36	1,14.54	0.62	0.445
	Light (off)	87.41 ± 15.94	1,286.45	30.06	9.20×10^{-08}
	Feeding (yes)	-49.10 ± 13.96	1,502.95	12.37	4.75×10^{-04}
	Cortisol (per 1 ng/ml)	-3.12 ± 0.45	1,251.12	47.45	4.52×10^{-11}
	Activity (per 1%)	-1.16 ± 0.22	1,425.58	29.22	1.08×10^{-07}
	Hematocrit (per 1%)	18.75 ± 2.78	1,346.86	45.39	6.72×10^{-11}
	Sampling (per sample)	2.55 ± 1.36	1,117.19	3.53	0.063

TABLE S2 | (*continued*)

Variable	Model term	Fixed coefficient ± SE	*df* [b]	*F*	*P*
Total Th cells[f]	*Corrected model* (Coefficient: Intercept)	1352.46 ± 202.29	7,82.56	32.69	$<1.00 \times 10^{-36}$
	Treatment (LD)	57.94 ± 177.13	1,18.06	0.11	0.747
	Light (off)	110.05 ± 21.39	1,449.23	26.46	4.03×10^{-07}
	Feeding (yes)	-7.59 ± 23.93	1,435.16	0.10	0.751
	Cortisol (per 1 ng/ml)	-3.00 ± 0.66	1,442.87	20.37	8.20×10^{-06}
	Activity (per 1%)	-3.38 ± 0.36	1,409.43	89.67	$<1.00 \times 10^{-36}$
	Hematocrit (per 1%)	28.33 ± 4.67	1,446.79	36.81	2.78×10^{-09}
	Sampling (per sample)	-3.59 ± 1.63	1,88.38	4.86	0.030
Cytotoxic T cells[d]	*Corrected model* (Coefficient: Intercept)	6.7845 ± 0.1351	7,512.00	38.51	$<1.00 \times 10^{-36}$
	Treatment (LD)	-0.1214 ± 0.1140	1,17.87	1.13	0.301
	Light (off)	0.1012 ± 0.0152	1,450.62	44.55	7.32×10^{-11}
	Feeding (yes)	-0.0046 ± 0.0164	1,434.37	0.08	0.780
	Cortisol (per 1 ng/ml)	-0.0012 ± 0.0005	1,461.24	6.77	0.010
	Activity (per 1%)	-0.0023 ± 0.0002	1,399.86	89.68	$<1.00 \times 10^{-36}$
	Hematocrit (per 1%)	0.0093 ± 0.0032	1,443.22	8.62	0.003
	Sampling (per sample)	-0.0020 ± 0.0012	1,84.02	3.12	0.081
Total γδ T cells[c]	*Corrected model* (Coefficient: Intercept)	7.7475 ± 0.1097	7,512.00	38.64	$<1.00 \times 10^{-36}$
	Treatment (LD)	0.1160 ± 0.1302	1,18.02	0.79	0.385
	Light (off)	0.0801 ± 0.0091	1,482.82	76.57	$<1.00 \times 10^{-36}$
	Feeding (yes)	-0.0027 ± 0.0097	1,425.04	0.08	0.782
	Cortisol (per 1 ng/ml)	-0.0013 ± 0.0003	1,492.50	21.84	3.82×10^{-06}
	Activity (per 1%)	-0.0012 ± 0.0001	1,383.31	68.81	1.78×10^{-15}
	Hematocrit (per 1%)	0.0141 ± 0.0019	1,409.08	56.13	4.23×10^{-13}
	Sampling (per sample)	-0.0005 ± 0.0009	1,48.30	0.38	0.542
Naive Th cells[c]	*Corrected model* (Coefficient: Intercept)	6.5871 ± 0.1243	7,512.00	62.26	$<1.00 \times 10^{-36}$
	Treatment (LD)	-0.0438 ± 0.1085	1,18.06	0.16	0.691
	Light (off)	0.1075 ± 0.0128	1,402.05	71.00	6.66×10^{-16}
	Feeding (yes)	-0.0054 ± 0.0148	1,405.23	0.13	0.717
	Cortisol (per 1 ng/ml)	-0.0032 ± 0.0004	1,364.31	67.52	3.77×10^{-15}
	Activity (per 1%)	-0.0024 ± 0.0002	1,386.15	118.91	$<1.00 \times 10^{-36}$
	Hematocrit (per 1%)	0.0174 ± 0.0029	1,442.55	36.68	2.97×10^{-09}
	Sampling (per sample)	-0.0007 ± 0.0009	1,74.23	0.57	0.454
Ag-exp. Th cells[c]	*Corrected model* (Coefficient: Intercept)	6.5419 ± 0.1031	7,512.00	8.32	1.22×10^{-09}
	Treatment (LD)	0.0783 ± 0.0927	1,18.06	0.71	0.409
	Light (off)	-0.0179 ± 0.0108	1,461.98	2.72	0.100
	Feeding (yes)	-0.0210 ± 0.0120	1,445.16	3.05	0.082
	Cortisol (per 1 ng/ml)	0.0007 ± 0.0003	1,461.39	3.89	0.049
	Activity (per 1%)	-0.0006 ± 0.0002	1,420.31	10.66	0.001
	Hematocrit (per 1%)	0.0099 ± 0.0023	1,451.34	17.73	3.08×10^{-05}
	Sampling (per sample)	-0.0028 ± 0.0008	1,97.76	10.53	0.002
$CD8\alpha^{-}$ γδ T cells[c]	*Corrected model* (Coefficient: Intercept)	7.4562 ± 0.1299	7,512.00	45.25	$<1.00 \times 10^{-36}$
	Treatment (LD)	0.1072 ± 0.1597	1,18.02	0.45	0.511
	Light (off)	0.0948 ± 0.0099	1,464.60	91.01	$<1.00 \times 10^{-36}$
	Feeding (yes)	-0.0059 ± 0.0107	1,410.83	0.30	0.583
	Cortisol (per 1 ng/ml)	-0.0015 ± 0.0003	1,477.62	23.73	1.51×10^{-06}
	Activity (per 1%)	-0.0014 ± 0.0002	1,366.67	74.84	2.22×10^{-16}
	Hematocrit (per 1%)	0.0128 ± 0.0021	1,401.26	37.55	2.13×10^{-09}
	Sampling (per sample)	-0.0005 ± 0.0009	1,47.08	0.36	0.551

TABLE S2 | (*continued*)

Variable	Model term	Fixed coefficient ± SE	*df* [b]	*F*	*P*
CD8α[+] γδ T cells[f]	*Corrected model* (Coefficient: Intercept)	475.23 ± 135.74	7,81.83	16.38	2.65×10^{-13}
	Treatment (LD)	129.60 ± 142.34	1,18.02	0.83	0.375
	Light (off)	56.78 ± 12.87	1,465.45	19.47	1.27×10^{-05}
	Feeding (yes)	11.02 ± 14.08	1,440.03	0.61	0.434
	Cortisol (per 1 ng/ml)	-1.83 ± 0.40	1,469.84	20.93	6.10×10^{-06}
	Activity (per 1%)	-0.90 ± 0.21	1,410.79	18.43	2.20×10^{-05}
	Hematocrit (per 1%)	18.23 ± 2.75	1,440.69	43.93	9.95×10^{-11}
	Sampling (per sample)	-1.07 ± 1.05	1,82.33	1.04	0.310

[a] y_{ij} = μ + $treatment_i$ + $light_{ij}$ + concentrate $feeding_j$ + plasma cortisol $concentration_{ij}$ + relative activity $behavior_{ij}$ + $hematocrit_{ij}$ + $sampling_j$ + animal $identity_i$ + ε_{ij}; dependent variable: y_{ij} (cell number/µl blood) for an animal *i* at sampling *j*; fixed effects: intercept μ, treatment (LD/SD), light (off/on), concentrate feeding (yes/no), plasma cortisol concentration (ng/ml), relative activity behavior (%), hematocrit (%), and sampling (1–26); random effect (with scaled identity (ID) covariance structure): animal identity (n = 20); repeated effect (with first order autoregressive (AR(1)) residual (ε_{ij}) covariance structure): sampling; subject: animal identity

[b] Numerator degrees of freedom, denominator degrees of freedom

[c] Gamma distribution with log link function

[d] Inverse Gaussian distribution with log link function

[e] Inverse Gaussian distribution with identity link function

[f] Normal distribution with identity link function

TABLE S3 | Results of overall cosinor analyses for CD8α⁻ and CD8α⁺ γδ T cells with combined datasets of all animals held under long day conditions (LD, n = 9) or short day conditions (SD, n = 11)

Variable	Treatment	*P*[a]	Mesor	Amplitude	Amplitude [%][b]	Peak time[c]
CD8α⁻ γδ T cells [/µl]	LD	<0.001	3058.1 ± 58.1	342.0 ± 79.5	11.2 ± 2.6	22:53 ± 00:57
	SD	<0.001	2851.3 ± 59.6	396.6 ± 82.5	13.9 ± 2.9	21:28 ± 00:50
CD8α⁺ γδ T cells [/µl]	LD	0.011	1191.2 ± 23.3	82.2 ± 32.4	6.9 ± 2.7	20:51 ± 01:33
	SD	<0.001	1056.8 ± 18.1	106.0 ± 25.6	10.0 ± 2.4	20:03 ± 00:55

Values are presented as mean ± SEM.
[a] Significant diurnal rhythm at $P < 0.05$
[b] Relative amplitude (amplitude/mesor)
[c] Time of day ± hh:mm

TABLE S4 | Summary of generalized linear mixed models for CD8α⁻ and CD8α⁺ γδ T cells[a]

Variable	Fixed effects: Estimated associations[b] with significance levels[c]						
	Treatment (LD)	Light (off)	Feeding (yes)	Cortisol	Activity	Hematocrit	Sampling
CD8α⁻ γδ T cells	↔	↑ ***	↔	↓ ***	↓ ***	↑ ***	↔
CD8α⁺ γδ T cells	↔	↑ ***	↔	↓ ***	↓ ***	↑ ***	↔

[a] Refer to Table S2 in Supplementary Material for details.
[b] Estimated association: ↑ positive, ↓ negative, ↔ none
[c] *** $P < 0.001$

TABLE S5 | Statistical details for pairwise comparisons of mesor values of the investigated variables between animals held under long day conditions (LD) or short day conditions (SD)

Variable	Treatment	n[a]	Statistics	Mean difference ± SEM	CI[b] or Pctl[c]
Leukocytes [/µl] [d]	LD	7	$t(16) = 0.92$, $P = 0.369$	1058.4 ± 1145.1	CI 14709.0–17720.1
	SD	11			CI 13330.6–16981.7
NK cells [/µl] [e]	LD	9	$U = 43$, $Z = -0.49$, $P = 0.621$	−1.6 ± 17.8	Pctl 64.8–122.4
	SD	11			Pctl 66.9–123.6
T cells [/µl] [d]	LD	9	$t(18) = 0.50$, $P = 0.623$	265.1 ± 529.4	CI 6838.0–8258.9
	SD	11			CI 6378.5–8188.1
Monocytes [/µl] [d]	LD	5	$t(14) = -0.34$, $P = 0.736$	−52.5 ± 152.6	CI 789.4–1041.6
	SD	11			CI 747.2–1188.7
Neutrophils [/µl] [d]	LD	9	$t(17) = 0.80$, $P = 0.437$	354.4 ± 444.8	CI 3071.5–4822.3
	SD	10			CI 3030.5–4154.5
Eosinophils [/µl] [d]	LD	9	$t(18) = 0.92$, $P = 0.368$	112.2 ± 121.6	CI 579.9–962.0
	SD	11			CI 466.0–851.4
Total Th cells [/µl] [e]	LD	8	$U = 42$, $Z = -0.17$, $P = 0.869$	130.8 ± 177.4	Pctl 1903.8–2828.3
	SD	11			Pctl 2002.0–2327.7
Cytotoxic T cells [/µl] [e]	LD	9	$U = 33$, $Z = -1.25$, $P = 0.210$	−155.0 ± 145.2	Pctl 858.1–1112.1
	SD	11			Pctl 973.2–1541.7
Total γδ T cells [/µl] [d]	LD	9	$t(18) = 0.68$, $P = 0.503$	341.3 ± 499.0	CI 3492.3–5006.5
	SD	11			CI 3101.3–4714.9
Naive Th cells [/µl] [e]	LD	9	$U = 35$, $Z = -1.10$, $P = 0.271$	−55.5 ± 136.6	Pctl 977.5–1360.8
	SD	11			Pctl 998.9–1503.8
Ag-exp. Th cells [/µl] [e]	LD	6	$U = 13$, $Z = -1.14$, $P = 0.253$	186.4 ± 163.9	Pctl 907.0–1264.1
	SD	7			Pctl 840.9–1003.1
$CD8\alpha^-$ γδ T cells [/µl] [d]	LD	9	$t(18) = 0.47$, $P = 0.645$	206.8 ± 441.3	CI 2354.0–3762.3
	SD	11			CI 2158.3–3544.3
$CD8\alpha^+$ γδ T cells [/µl] [d]	LD	9	$t(18) = 0.90$, $P = 0.380$	134.5 ± 149.4	CI 913.1–1469.3
	SD	11			CI 850.6–1262.9
Cortisol [ng/ml] [d]	LD	9	$t(18) = -0.10$, $P = 0.921$	−0.1 ± 1.5	CI 13.4–17.4
	SD	11			CI 13.0–18.1
Activity behavior [%] [f]	LD	9	$t(13.19) = -1.24.$, $P = 0.236$	−2.0 ± 1.6	CI 19.6–25.9
	SD	11			CI 22.9–26.5
Hematocrit [%] [d]	LD	8	$t(17) = 2.28.$, $P = 0.036$	1.3 ± 0.6	CI 33.6–35.2
	SD	11			CI 32.1–34.0

[a] Number of animals out of 9 for LD or out of 11 for SD, which were included into pairwise comparisons (animals with significant ($P < 0.05$) diurnal rhythm in individual cosinor analyses, refer to Table S1 in Supplementary Material)
[b] 95% confidence interval
[c] 25th–75th percentile
[d] Unpaired Student's *t*-test (two-tailed)
[e] Mann-Whitney *U* test (two-tailed)
[f] Unequal variance *t*-test (two-tailed)

TABLE S6 | Statistical details for pairwise comparisons of relative amplitudes of the investigated variables between animals held under long day conditions (LD) or short day conditions (SD)

Variable	Treatment	n[a]	Statistics	Mean difference ± SEM [%]	CI[b] or Pctl[c] [%]
Leukocytes [/µl] [e]	LD	7	U = 4, Z = −3.13, P = 0.002	−2.9 ± 0.9	Pctl 5.5–6.8
	SD	11			Pctl 7.7–9.0
NK cells [/µl] [d]	LD	9	t(18) = −2.54, P = 0.021	−10.6 ± 4.2	CI 26.1–41.2
	SD	11			CI 38.3–50.1
T cells [/µl] [e]	LD	9	U = 19, Z = −2.32, P = 0.020	−3.3 ± 1.4	Pctl 7.2–11.2
	SD	11			Pctl 10.5–13.4
Monocytes [/µl] [d]	LD	5	t(14) = −3.14., P = 0.007	−4.6 ± 1.5	CI 5.7–11.0
	SD	11			CI 11.0–14.9
Neutrophils [/µl] [d]	LD	9	t(17) = −0.96, P = 0.348	−1.5 ± 1.5	CI 9.9–15.6
	SD	10			CI 12.1–16.3
Eosinophils [/µl] [e]	LD	9	U = 26, Z = −1.79, P = 0.074	−5.0 ± 2.5	Pctl 27.9–35.4
	SD	11			Pctl 32.8–41.1
Total Th cells [/µl] [d]	LD	8	t(17) = −1.61, P = 0.126	−2.1 ± 1.3	CI 5.8–9.6
	SD	11			CI 7.7–12.0
Cytotoxic T cells [/µl] [d]	LD	9	t(18) = −1.02, P = 0.320	−2.6 ± 2.6	CI 10.6–17.5
	SD	11			CI 12.3–21.0
Total γδ T cells [/µl] [d]	LD	9	t(18) = −2.02, P = 0.058	−2.6 ± 1.3	CI 8.4–11.7
	SD	11			CI 10.4–15.0
Naive Th cells [/µl] [e]	LD	9	U = 20, Z = −2.24, P = 0.025	−3.2 ± 1.4	Pctl 12.6–16.6
	SD	11			Pctl 15.4–18.0
Ag-exp. Th cells [/µl] [e]	LD	6	U = 16, Z = −0.71, P = 0.475	0.1 ± 2.5	Pctl 4.0–12.3
	SD	7			Pctl 5.3–10.3
$CD8\alpha^-$ γδ T cells [/µl] [d]	LD	9	t(18) = −1.87., P = 0.078	−2.6 ± 1.4	CI 9.8–13.3
	SD	11			CI 11.7–16.5
$CD8\alpha^+$ γδ T cells [/µl] [d]	LD	9	t(18) = −1.34, P = 0.197	−1.9 ± 1.4	CI 7.4–10.5
	SD	11			CI 8.3–13.5
Cortisol [ng/ml] [e]	LD	9	U = 43, Z = −0.49, P = 0.621	−2.4 ± 6.8	Pctl 84.7–108.7
	SD	11			Pctl 94.8–108.1
Activity behavior [%] [d]	LD	9	t(18) = −8.03, $P = 2.32 \times 10^{-07}$	−44.8 ± 5.6	CI 59.6–82.9
	SD	11			CI 109.6–122.6
Hematocrit [%] [d]	LD	8	t(17) = 0.13, P = 0.902	0.1 ± 0.7	CI 4.0–6.2
	SD	11			CI 4.0–6.0

[a] Number of animals out of 9 for LD or out of 11 for SD, which were included into pairwise comparisons (animals with significant (P < 0.05) diurnal rhythm in individual cosinor analyses, refer to Table S1 in Supplementary Material)
[b] 95% confidence interval
[c] 25th–75th percentile
[d] Unpaired Student's t-test (two-tailed)
[e] Mann-Whitney U test (two-tailed)

TABLE S7 | Statistical details for pairwise comparisons of peak times of the investigated variables between animals held under long day conditions (LD) or short day conditions (SD)

Variable	Treatment	n[a]	Statistics	Mean difference ± SEM [hh:mm]	CI[b] or Pctl[c] [time of day]	StdDev[g] [hh:mm]
Leukocytes [/µl] [d]	LD	7	$t(16) = 3.17$, $P = 0.006$	02:42 ± 00:51	CI 19:35–23:51	02:18
	SD	11			CI 18:08–19:55	01:20
NK cells [/µl] [d]	LD	9	$t(18) = 5.41$, $P = 3.85 \times 10^{-05}$	02:20 ± 00:26	CI 12:17–14:03	01:09
	SD	11			CI 10:19–11:21	00:46
T cells [/µl] [f]	LD	9	$t(11.33) = 4.03$, $P = 0.002$	01:24 ± 00:21	CI 22:02–23:29	00:57
	SD	11			CI 21:02–21:41	00:29
Monocytes [/µl] [f]	LD	5	$t(5.13) = 1.37$, $P = 0.229$	01:55 ± 01:24	CI 15:44–23:02	02:56
	SD	11			CI 16:23–18:33	01:37
Neutrophils [/µl] [e]	LD	9	$U = 27$, $Z = -1.47$, $P = 0.142$	−01:35 ± 00:50	Pctl 12:15–13:20	02:15
	SD	10			Pctl 12:44–14:54	01:20
Eosinophils [/µl] [d]	LD	9	$t(18) = 7.08$, $P = 1.34 \times 10^{-06}$	02:12 ± 00:19	CI 21:25–22:42	00:50
	SD	11			CI 19:28–20:13	00:33
Total Th cells [/µl] [d]	LD	8	$t(17) = 1.40$, $P = 0.179$	01:00 ± 00:43	CI 20:12–23:52	02:11
	SD	11			CI 20:29–21:34	00:48
Cytotoxic T cells [/µl] [d]	LD	9	$t(18) = 5.95$, $P = 1.26 \times 10^{-05}$	02:31 ± 00:25	CI 23:49–01:34	01:08
	SD	11			CI 21:41–22:41	00:45
Total γδ T cells [/µl] [d]	LD	9	$t(18) = 3.97$, $P = 8.92 \times 10^{-04}$	01:19 ± 00:20	CI 21:45–23:08	00:54
	SD	11			CI 20:43–21:31	00:35
Naive Th cells [/µl] [e]	LD	9	$U = 2$, $Z = -3.61$, $P = 3.08 \times 10^{-04}$	01:41 ± 00:26	Pctl 22:10–00:27	01:15
	SD	11			Pctl 21:09–21:36	00:19
Ag-exp. Th cells [/µl] [d]	LD	6	$t(11) = -0.30$, $P = 0.767$	−00:51 ± 02:47	CI 10:03–19:05	04:18
	SD	7			CI 10:18–20:32	05:32
CD8α^- γδ T cells [/µl] [d]	LD	9	$t(18) = 5.51$, $P = 3.12 \times 10^{-05}$	01:25 ± 00:16	CI 22:26–23:26	00:39
	SD	11			CI 21:10–21:51	00:30
CD8α^+ γδ T cells [/µl] [d]	LD	9	$t(18) = 0.61$, $P = 0.547$	00:37 ± 01:01	CI 17:57–22:18	02:50
	SD	11			CI 18:24–20:36	01:38
Cortisol [ng/ml] [d]	LD	9	$t(18) = 5.60$, $P = 2.60 \times 10^{-05}$	01:44 ± 00:19	CI 07:45–08:52	00:44
	SD	11			CI 06:08–07:01	00:40
Activity behavior [%] [f]	LD	9	$t(12.23) = 6.86$, $P = 1.58 \times 10^{-05}$	02:39 ± 00:23	CI 14:25–16:00	01:02
	SD	11			CI 12:09–12:57	00:36
Hematocrit [%] [d]	LD	8	$t(17) = 3.32$, $P = 0.004$	01:16 ± 00:23	CI 14:11–15:32	00:48
	SD	11			CI 13:03–14:09	00:49

[a] Number of animals out of 9 for LD or out of 11 for SD, which were included into pairwise comparisons (animals with significant ($P < 0.05$) diurnal rhythm in individual cosinor analyses, refer to Table S1 in Supplementary Material)
[b] 95% confidence interval
[c] 25th–75th percentile
[d] Unpaired Student's *t*-test (two-tailed)
[e] Mann-Whitney *U* test (two-tailed)
[f] Unequal variance *t*-test (two-tailed)
[g] Standard deviation of mean peak times to define phase distribution

TABLE S8 | Statistical details for pairwise comparisons of mean values of the investigated variables between animals held under long day conditions (LD) or short day conditions (SD)

Variable	Treatment	n[a]	Statistics	Mean difference ± SEM	CI[b] or Pctl[c]
Leukocytes [/µl] [d]	LD	9	$t(18) = 0.40$, $P = 0.693$	429.9 ± 1071.9	CI 14032.5–17028.6
	SD	11			CI 13298.2–16903.2
NK cells [/µl] [e]	LD	9	$U = 43$, $Z = -0.49$, $P = 0.621$	−2.6 ± 18.4	Pctl 65.7–125.2
	SD	11			Pctl 68.8–126.9
T cells [/µl] [d]	LD	9	$t(18) = 0.53$, $P = 0.603$	276.7 ± 523.4	CI 6793.5–8202.4
	SD	11			CI 6327.8–8114.6
Monocytes [/µl] [d]	LD	9	$t(18) = -0.22$, $P = 0.826$	−26.2 ± 117.9	CI 826.8–1053.5
	SD	11			CI 747.2–1185.5
Neutrophils [/µl] [d]	LD	9	$t(18) = 0.42$, $P = 0.678$	195.0 ± 462.0	CI 3094.7–4855.8
	SD	11			CI 3160.1–4400.5
Eosinophils [/µl] [d]	LD	9	$t(18) = 0.91$, $P = 0.376$	108.0 ± 119.0	CI 565.7–941.5
	SD	11			CI 457.4–833.8
Total Th cells [/µl] [e]	LD	9	$U = 42$, $Z = -0.57$, $P = 0.569$	69.0 ± 176.1	Pctl 1873.5–2615.2
	SD	11			Pctl 1978.6–2314.6
Cytotoxic T cells [/µl] [e]	LD	9	$U = 33$, $Z = -1.25$, $P = 0.210$	−149.7 ± 142.4	Pctl 854.4–1098.3
	SD	11			Pctl 967.5–1522.5
Total γδ T cells [/µl] [d]	LD	9	$t(18) = 0.69$, $P = 0.496$	343.5 ± 494.7	CI 3466.4–4971.2
	SD	11			CI 3076.3–4674.3
Naive Th cells [/µl] [e]	LD	9	$U = 35$, $Z = -1.10$, $P = 0.271$	−53.3 ± 135.1	Pctl 969.4–1345.6
	SD	11			Pctl 983.1–1490.0
Ag-exp. Th cells [/µl] [e]	LD	9	$U = 37$, $Z = -0.95$, $P = 0.342$	122.3 ± 112.8	Pctl 836.7–1032.7
	SD	11			Pctl 825.4–999.1
$CD8\alpha^-$ γδ T cells [/µl] [d]	LD	9	$t(18) = 0.48$, $P = 0.640$	208.6 ± 437.9	CI 2333.4–3732.1
	SD	11			CI 2137.1–3511.3
$CD8\alpha^+$ γδ T cells [/µl] [d]	LD	9	$t(18) = 0.91$, $P = 0.374$	134.9 ± 148.1	CI 910.2–1461.9
	SD	11			CI 846.9–1255.3
Cortisol [ng/ml] [d]	LD	9	$t(18) = 0.16$, $P = 0.879$	0.2 ± 1.5	CI 14.1–18.3
	SD	11			CI 13.4–18.6
Activity behavior [%] [f]	LD	9	$t(13.42) = -1.89$, $P = 0.081$	−3.3 ± 1.8	CI 19.9–26.8
	SD	11			CI 24.6–28.6
Hematocrit [%] [d]	LD	9	$t(18) = 1.82$, $P = 0.085$	1.1 ± 0.6	CI 33.3–35.1
	SD	11			CI 32.2–34.1

[a] Data of all animals per treatment were included in comparisons.
[b] 95% confidence interval
[c] 25th–75th percentile
[d] Unpaired Student's *t*-test (two-tailed)
[e] Mann-Whitney *U* test (two-tailed)
[f] Unequal variance *t*-test (two-tailed)

TABLE S9 | Statistical details for mixed ANOVA investigating the effects of treatment (long day conditions (LD)/short day conditions (SD)) and light (on/off)[a]

Variable	Model term	Pairwise comparisons	Mean difference ± SEM	*Partial* η^2	*df* [b]	*F*	*P*
Cortisol [ng/ml]	Treatment			<0.01	1,18	0.77	0.785
	Light			0.48	1,18	16.53	7.25×10^{-04}
	Treatment × Light			0.63	1,18	30.44	3.08×10^{-05}
	Simple main effects:	LD – on *vs.* off	1.2 ± 1.2	0.05	1,18	0.96	0.341
		SD – on *vs.* off	−7.7 ± 1.1	0.74	1,18	51.02	1.18×10^{-06}
		On – SD *vs.* LD	4.9 ± 1.9	0.26	1,18	6.21	0.023
		Off – SD *vs.* LD	−4.0 ± 1.5	0.29	1,18	7.19	0.015
Activity behavior [%]	Treatment			0.78	1,18	64.46	2.33×10^{-07}
	Light			0.95	1,18	352.76	2.84×10^{-13}
	Treatment × Light			0.43	1,18	13.54	0.002
	Simple main effects:	LD – on *vs.* off	−25.7 ± 2.5	0.85	1,18	103.67	6.76×10^{-09}
		SD – on *vs.* off	−38.2 ± 2.3	0.94	1,18	280.29	2.02×10^{-12}
		On – SD *vs.* LD	18.9 ± 3.2	0.66	1,18	35.54	1.22×10^{-05}
		Off – SD *vs.* LD	6.4 ± 0.8	0.77	1,18	59.41	4.14×10^{-07}
Hematocrit [%]	Treatment			0.02	1,18	0.39	0.540
	Light			0.93	1,18	245.71	6.16×10^{-12}
	Treatment × Light			0.05	1,18	0.95	0.342

[a] 2 × 2 mixed factorial ANOVA with treatment (LD/SD) as between-subjects factor and light (on/off) as within-subjects repeated factor was performed on mean values calculated from the 26 samplings per pig, divided into values during lights-on or lights-off for all animals per treatment (LD n = 9, lights-on = 18 samplings & lights-off = 8 samplings, SD n = 11, lights-on = 10 samplings & lights-off = 16 samplings). If there was a significant interaction effect, simple main effects were analyzed with the least significant difference (LSD) procedure.
[b] Numerator degrees of freedom, denominator degrees of freedom
